# Subsidies versus mental accounting nudges: Harnessing mobile payment systems to improve sanitation^☆^

**DOI:** 10.1016/j.jdeveco.2018.07.007

**Published:** 2018-11

**Authors:** Molly Lipscomb, Laura Schechter

**Affiliations:** a UVA, USA; b UW Madison, USA

**Keywords:** Earmarking, Mental accounting, Mobile money, Sanitation, Savings, Subsidies

## Abstract

The proliferation of mobile money across developing countries has led to an increase in availability of mobile payment systems. This decreases the organizational complexity of allowing more flexible payment terms for customers. We test whether subsidies, deposit requirements, and access to a mobile money savings vehicle increase the propensity of households to purchase an improved but more expensive sanitation service. While high subsidies increase purchases of the improved service, interventions inspired by mental accounting such as deposit requirements and earmarked savings accounts do not. The option to save in earmarked accounts using mobile money caused households to substitute away from purchasing the improved service in the general market and towards purchasing it through our providers, rather than substituting away from the unimproved service. We discuss implications for mental accounting-based policies compared to more traditional subsidies.

## Highlights

• Subsidies increase adoption of sanitation; mental accounting nudges do not.
• Requiring deposits reduces sign-up by 10%, but has no overall effect on take-up.
• Consumers value earmarked accounts and switch to suppliers who provide them.
• Earmarked accounts are most valued by people who would have purchased anyway.

## 1 Introduction

Low demand for health-enhancing products and services imposes substantial welfare costs on communities as health and sanitation goods have large spillover effects. Households tend to be price elastic in their demand for health-enhancing technologies (Ashraf et al., 2010; Cohen and Dupas, 2010; Kremer and Miguel, 2007). In addition, households may have larger willingness to pay than ability to pay as a result of liquidity constraints and the difficulty of saving or borrowing for these items (Dupas and Robinson, 2013; Guiteras et al., 2016; Tarozzi et al., 2014). It has been widely shown that large subsidies can increase the take-up of these goods (Bates et al., 2012), but subsidy programs are expensive. We analyze the potential for mobile payment systems to increase take-up of sanitation services through interventions aimed at relaxing budget constraints and liquidity constraints.

More flexible payment plans involving credit are one way to increase take-up and have worked in other settings. Ben Yishay et al. (2017) finds that the willingness to pay for latrines increases substantially when households are offered the chance to pay for the latrine over time. In our setting, the sanitation technology is a service rather than a durable, so lending is more difficult: after the service is provided, there is no collateral to encourage households to repay their loans. Because studies such as Guiteras et al. (2016) and Afzal et al. (2017) find that borrowing and saving are substitutes, we focus on relaxing liquidity constraints through saving interventions rather than borrowing interventions.

Access to financial products allowing households to save remains a major problem for households in developing countries (Dupas et al., 2018; Karlan et al., 2014), but has improved substantially through increased access to mobile money (Suri and Jack, 2016). While MPESA has been found to provide informal insurance and reduce exposure to risk in Kenya (Jack and Suri, 2014; Suri et al., 2012), mobile money has quickly become more versatile and has been used with increasing frequency to make payments to workers and households (Blumenstock et al., 2015; Brune et al., 2016). To our knowledge, there is less research available on using mobile money to facilitate installment payments for specific goods. Allowing individuals to make partial payments in advance in earmarked accounts, or forcing them to do so by requiring pre-paid deposits, could increase purchases of the good. This is both because having an account gives a household a place to save money and because the earmarking and mandatory deposit encourage mental accounting.

Mental accounting models suggest that households have several spending categories and only allow themselves to make a purchase when they have available funds targeted to that category (Thaler, 1985). Such models would predict that providing households with accounts earmarked for a specific purpose will increase the amount of spending households dedicate to that use. Households may also be more likely to purchase a product if they feel that they have already invested a “sunk cost” (Thaler, 1999). Requiring households to pay a deposit in order to reserve their improved sanitation service could serve as a sunk cost and increase purchases.

Commitment savings mechanisms have been implemented to improve households’ ability to save for important but infrequent expenditures, but they have had mixed success in a variety of settings (Ashraf et al., 2006; Blumenstock et al., 2018; Brune et al., 2016; Dupas and Robinson, 2013; Karlan and Linden, 2014; Kast et al., 2012). Dupas and Robinson (2013) finds that earmarked savings mechanisms help individuals save for health emergencies, but are much less effective at helping people save for the type of preventative health purchases that we study. Mobile money may be one way to facilitate commitment savings. Habyarimana and Jack (2018) evaluates the use of mobile money to increase savings for high school education expenses. Similar to our results, they find no additional benefit from having an earmarked locked account over having a non-earmarked account.

In this paper, we test the relative impact of mental accounting nudges to increase savings through mobile payment systems versus subsidies in increasing the take-up of sanitation products. Households in Dakar, which are off of the networked sewage lines, need to purchase this service approximately once every six months.^1^ They can choose a manual desludging which is cheaper but less sanitary, or a mechanized desludging which is more expensive but more sanitary. We test the impact of mental accounting nudges such as earmarked savings and sunk cost deposits relative to more traditional subsidies on the service.

We offer households the opportunity to sign up in advance for a subsidized mechanized desludging subscription with subsidies randomized between two levels. We randomize components of the payment process in order to measure the impact of mental accounting nudges. In order to test the importance of deposits, we randomize the requirement that households make a deposit of $6 (either 12.5% or 17.6% of the full price depending on their subsidy level) toward the purchase price in order to sign up for access to the service during the baseline survey.

In order to test whether mental accounting nudges can help households save for the desludging, we randomize whether the desludging account will accept deposits of amounts less than the full price. The group which is allowed to make non-final deposits is further randomized into two groups: a group that is asked to make consistent partial advance payments each month (monthly billing), and a group that is asked to save whenever they have available funds (save at will). The monthly-billing system was meant to help clients purchase a desludging through nudging them toward consistent monthly payments equivalent to the average smoothed costs of desludging services over a year. The save-at-will treatment was meant to allow households maximum flexibility in saving for the service. Control group households (pay in full) are not able to deposit partial payments into their accounts. This approximates the status quo system in which a household pays the desludging operator in full for their work at the time of service. All households receive monthly reminders of the program and the availability of the desludging adapted to their treatment group and must have deposited the full price of their desludging prior to receiving the service.

We find that subsidies do encourage households to switch to more sanitary desludging services. Households are eight percentage points more likely to purchase a mechanized desludging from the program and three percentage points more likely to purchase a mechanized desludging overall when offered a large subsidy relative to a small subsidy. On the other hand, earmarked accounts, pre-paid deposits, and monthly billing do not have an impact on overall sanitation purchases. There are a few potential reasons for this divergence from the predictions of mental accounting theories. First, desludging purchases are infrequent and somewhat unpredictable expenses, which may make tracking them as a dedicated mental account more difficult. Second, targeting funds to a specific spending category may be more useful for some types of consumers. We only randomize access to the earmarked savings accounts among individuals who first signed up for the subscription desludging service. It could be that earmarking money towards desludging purchases has a smaller effect on those who at baseline already plan to purchase the subsidized mechanized desludging (the subscribers). It is possible that these accounts might have had a larger impact acting as an added incentive for individuals who did not originally sign up for the desludging subscription.

While the earmarked mobile money accounts do not cause households to switch from manual to mechanized desludgings, they do cause households to purchase the subsidized desludging through us rather than on the open market. Households increase their purchases of our program desludgings by five percentage points and have a corresponding similarly sized decrease in their use of non-program desludgings. The reason why individuals in the save-at-will group are significantly more likely to purchase the subsidized service appears to be that they appreciate the ability to deposit amounts below the full price of the good, and the ability to save in anticipation over longer periods of time. Households in our save-at-will group wait nearly 50% longer between their first deposit and the use of the desludging than households in the pay-in-full group. The monthly-billing treatment has no such positive effect on purchases of the subsidized services, frequency of smaller deposits, and time spent saving in anticipation of purchase. This suggests that the save-at-will treatment gives people a way to earmark their money for a specific service, and that individuals prefer the flexibility in payment terms that the save-at-will treatment offers them but the monthly-billing treatment does not.

We explore heterogeneous effects of the subsidy and savings interventions across those with risky and those with stable incomes. We also look at heterogeneous impacts across those with different mechanized desludging histories. Subsidies increase the use of more sanitary desludging techniques and this is especially true for individuals whose sanitation decisions may be most influenced by the price of a desludging: those with a salaried job and those who have purchased a mechanized desludging in the past but not in the year before the baseline.

The different mobile money and deposit treatments which attempt to take advantage of mental accounting do not change general sanitation purchase behavior overall or for any subgroup. Yet, some people who were going to purchase a mechanized desludging anyway such as those who have used a mechanized desludging in the past year, and those who need more help saving such as those without a regular paying job, do at least seem to appreciate and take advantage of the opportunity to save at will in a mobile money account and switch from purchasing in the general market to purchasing through our service.

## 2 Background

While several papers have discussed sanitation issues in rural contexts (Coffey et al., 2017; Pattanayak et al., 2009), urban communities face different, but equally complex, issues (Hathi et al., 2017). Latrine and toilet ownership is common in urban areas, but the disposal of latrine waste can be problematic for those who are not on the sewage network. Improper removal and disposal of latrine waste is common and leads to important health repercussions (Mara et al., 2010).

Central downtown Dakar (the capital of Senegal) has a functioning sewage network. But, almost two million people in urban Dakar outside the city center use latrines which are not connected to the city's sewage network. These pits fill up approximately once every six months and then need to be emptied, or “desludged,” for continued use.^2^ When the latrine pit is full, households have two options: manual or mechanized desludging. In a manual desludging, a person enters the pit with a shovel and a bucket and dumps the sludge in the courtyard or in the street in front of the house. In our baseline survey, 56% of households chose this option for their most recent desludging. Of these, 53% of manual desludgings are done by a family member, usually for free (94% of the time); and 47% are done by a person hired for the task at an average price of $29. Households who had a family member conduct a manual desludging reported dumping the sludge in their own courtyard 34% of the time, in front of their house 32% of the time, in the street 24% of the time, and in a vacant lot 6% of the time. For those who hired a non-family member to provide the service, the dumping patterns were similar (21%, 38%, 29%, and 7% respectively). Forty-four percent of households chose mechanized desludging for their most recent desludging prior to the baseline. These households hired a truck to pump the sludge out of their pit and transport it to dump at a treatment center, for an average price of $50.

Many households choose manual desludgings due to the high price of mechanized desludgings. We asked households who had gotten desludged in the past but *never* purchased a mechanized desludging the primary reason they had not purchased a mechanized desludging. The high price was cited by 62%; 26% were concerned about their house not being accessible by the desludging truck (for example due to sandy or narrow roads); and 6% said that they heard rumors that trucks leave sludge in the pits.

Although desludging is a regular maintenance need, typically required between once and twice a year, many households do not plan their desludging until the pit is full. When pits need to be desludged, it often comes on as an urgent necessity. A household may want to purchase a mechanized desludging, but end up doing a manual desludging instead due to liquidity constraints in their moment of need. Of those who had their current pit desludged, 83% stated that they did so because the toilet was backing up, the pit was overflowing, or there were pests or an odor coming from the pit; 14% said they looked in the pit and saw it was getting full; while only 4% said it was a regularly scheduled or preventive action. Of those who had a desludging, 67% claimed they did it within two days of realizing they needed a desludging. For those who waited, 67% said that the reason they waited was lack of access to money and 16% said it was due to lack of access to labor. Thus liquidity appears to play a central role in households’ desludging decisions.

Individuals pay for mechanized desludgings in full in cash at the time of service. Credit is rarely given. It is usually relatively easy to find a desludger able to perform the service. As mentioned above, 67% of individuals purchased a desludging within two days of realizing they needed it. Of the select sample of those who waited more than two days, only 11% said that the reason they waited was that they had trouble finding a desludger.

Customer loyalty in the market seems to be relatively low. Most households who had purchased a mechanized desludging (61%) claim that their most recent desludging was performed by somebody who had not previously desludged for them. The first time a household used its most recent mechanized desludging operator 48% claim to have found the operator by getting a referral from a friend, 18% found the operator at their garage, 18% called a phone number they saw written on a truck, and 11% hailed the desludging truck down on the street.

### 2.1 Mobile money in Senegal

Dakar has three main mobile money service providers: Wari, Orange Money, and Joni-Joni. Wari is the largest provider in Dakar with 97% of respondents reporting that they knew of Wari and 86% stating that they had used Wari in the past; by contrast, 15% state that they had used Joni-Joni, and 13% state that they had used Orange Money. Although Wari is the primary mobile money provider in Dakar, their focus has been on transfers rather than savings. They did not offer mobile savings accounts before working with us. As of July 2014, Wari controlled 80% of the market for mobile money transfers in Senegal with an average of 125,000 daily remittances. Transactions are made through the 3000 Wari stations, typically located in corner shops (Williams, 2014).

Wari, like many money transfer services, offers services across Dakar by partnering with gas stations, internet cafes, and local corner stores. This provides them a much wider reach than a traditional bank, which may only have a few branches centered in wealthier areas of the city. Individuals can transfer money to other private individuals by visiting any Wari partner. A client makes a deposit at a Wari station, the transfer is recorded and tracked using the phone number of the recipient, and the recipient receives an SMS text message letting him know the money is available. The recipient can then go to any Wari station to pick up the funds. Individuals can also use Wari to pay their utility bills or receive pension payments.

Wari was interested in expanding their financial services and so, for the purposes of our project, they created Wari savings accounts and allowed our subscribers (and only our subscribers) to save money in them.^3^ At the time of our experiment, the only mobile money savings possibility was through Orange Money. Only 13% of our sample had used Orange Money for any service, and none of our sample had used an Orange Money savings account. This is partly because Wari was much more ubiquitous in the poorer urban areas in which our experiment was conducted, while Orange Money had very few transaction points in the neighborhoods in which we worked.

We will discuss our experiments in detail in the next section, but for comparison, the accounts we gave our participants required them to pay a $0.20 fee to deposit any amount less than $10 and a 2% fee for deposits larger than that amount. There were no fees for withdrawals or transfers to other accounts. Orange Money charges nothing to make deposits in their savings accounts, and approximately 6% on withdrawals.^4^ We chose to work with Wari because of its wide customer network, popularity, and willingness to work with us to integrate our experimental payment system into its existing system.

Brick and mortar banks also offer savings and checking accounts. While there is variety across institutions, in general for both types of accounts withdrawing is free but depositing is not. The main difference between savings and checking accounts is that most savings accounts have initial deposit requirements and/or minimum balance requirements but no maintenance fees, while checking accounts have no such requirements but do charge maintenance fees. Also, savings accounts may pay up to 3.5% interest while checking accounts do not. In sum, the fee structure on the Wari accounts we offered to the households in our study were better than the fees charged by other mobile money providers or by brick and mortar banks. In addition, it was much easier for participants to work with Wari due to their ubiquity.

## 3 Sampling and experimental design

Our target areas in Dakar are the areas most affected by manual desludging–the urban areas of the city to which the sanitation network does not extend. These households rely on stand-alone latrines. Sampling in an urban environment outside of the city center poses different challenges from those faced when sampling formal urban environments or rural environments. In a formal urban environment, municipal authorities may have a full listing of households which could be used for randomization. Conversely, in a rural environment, one could conduct a census of households in each of the locations. Dakar, similar to much of urban West Africa, is growing quickly and the government has few accurate lists of households located in neighborhoods outside of the city center.

**Fig. B-1:**
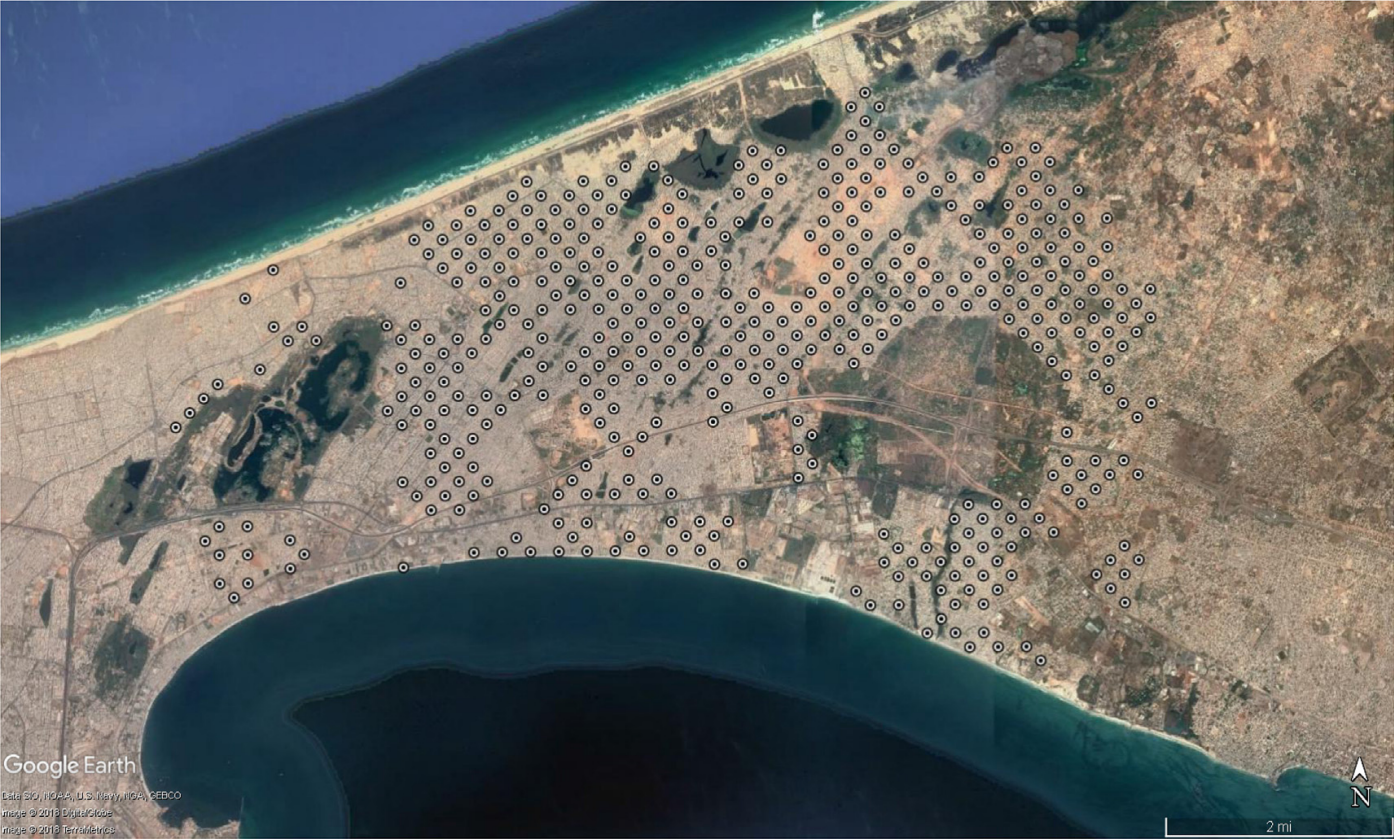
Map of gridpoints used as starting point for each neighborhood.

We were interested in balancing geographical representation with the logistical simplicity which comes when surveyed households are located more closely together. To do this, we placed a grid of points evenly spaced every 285 m across Dakar, and removed points which fell on parks, markets, military barracks, industrial areas, or areas already connected to the sewage network. This left us with 410 grid-points. The grid-points across Dakar are shown in Fig. B-1.

We created a precise rule determining which households would be surveyed to avoid giving enumerators discretion. In order to minimize selection bias, we mapped 25 households close to each grid-point using a standard formula.^5^ We then began approaching those 25 mapped households in a pre-specified randomly determined order until we found 12 who had a functioning pit and for which the household made the desludging decision (e.g., not a renter if the owner was the one in charge of desludging decisions).

A randomly selected ten of those were offered the subsidized desludging.^6^ Households could choose to sign up for the subscription which gave them access to up to two discounted mechanized desludgings over a period of 12 months. Among these households we randomized the subsidy level and the deposit requirement. The script which the enumerators used in the survey to introduce the subscription service can be found in Appendix A.

Because our survey showed that the average cost of an unsubsidized mechanized desludging is $50 and the average cost of a manual desludging not conducted by a family member is $29, we offered subsidized prices on mechanized desludgings, randomly offering half of the households a price of $48 and half a price of $34. This meant that the cost with the low subsidy is very close to that which the household would pay on the open market for a mechanized desludging, while the highly subsidized price is much closer to what the household would expect to pay for a cheaper, less sanitary, manual desludging.

All households received a $6 payment for their participation in the survey. Of these, 87% were randomly required to leave this in their Wari account as a deposit if they signed up for the subscription (but could access the money through Wari immediately if they did not sign up for the subscription). If they still had not used the subscription by the end of the 12 months, they were given access to their original $6 deposit and any funds saved in the account. The other 13% could sign up as purely cheap talk, with no commitment on their part.

The subsidy level and the deposit requirement randomizations were stratified at the neighborhood (grid-point) level. We first randomized how many households in each neighborhood would be offered the high subsidy (between one and nine out of ten) and how many would be required to pay a deposit to sign up (between six and ten out of ten). Then, within each neighborhood we randomized the subsidy and deposit requirements across households.^7^

Subscribing households were given a phone number they were told to call when they had saved enough money and wanted to use their subsidized desludging. This phone number was manned by two operators working for our research project. The operators asked the caller their member number or name and phone number, looked up the price that the individual was offered during the baseline survey, verified that the individual had enough money saved in his mobile money account, and procured a desludging from participating desludging operators.^8^ Upon completion of the desludging service, the operator transferred the payment from the household's account to the account of the desludging operator.^9^

Households were told that the money deposited in the account was committed to desludging services. Withdrawing the funds required the assistance of a project manager, so it was not possible for households to game the system by calling for the desludging while they had the funds in the account and then withdrawing the money before the service occurred, nor could they order the service and then refuse to pay. Subsidized desludgings could not be purchased by directly paying cash to the desludger.

In the baseline survey, households often claim to hire desludging operators they have used in the past or rely on recommendations from friends and relatives when using a new operator. With our subscription system, households are unable to request a specific desludging operator. The anonymity of our platform might lead households to worry about receiving inferior quality services compared with working with a firm that cares about retaining customers, but only 4% of households that did not use our service claimed it was because they wanted to use their usual provider. After households purchased desludgings through the subscription system, we followed up to solicit their opinion of the job done by the desludger. As 98% rated the work as high quality, 2% rated the work as medium quality, and only one individual said he was unsatisfied, we believe that the services we procured were at least as high quality as a typical desludging purchased in the market.

Of the 3757 households which were offered the subscription, 1496 enrolled.^10^ After making the enrollment decision, the 1496 households which signed up were randomized into one of three mobile money interventions. All 1496 subscribers received monthly SMS messaging, but the content of the message, and the functionality of their account, varied depending on the treatment group. We will call the three groups save at will, monthly billing, and pay in full, though these names were not mentioned to participants.

The pay-in-full treatment is closest to the current status quo, requiring the individual to pay in one installment. They may deposit that amount at any time, but in general they did so just before purchasing the service. The mobile money system prevented them from depositing any amount less than the full price. We treat this as the control group. The save-at-will treatment gives individuals a mechanism to save by providing them with a mobile money savings account earmarked for desludging expenses. They could deposit any amount at any time. Finally, the monthly-billing system was meant to give individuals an earmarked account in which to deposit payments. Encouraging clients to deposit the specific amount could smooth their payments over time.

The lumpy and unpredictable nature of desludging expenses may make it hard for households to plan. The median household needs a desludging approximately every 6 months, but a large proportion of households need desludgings substantially more often. The monthly-billing treatment was meant to mimic the features of ‘budget billing’ or ‘average monthly payment’ billing offered by many US utilities to allow consumers to smooth utility payments across months. Proponents of bill smoothing systems suggest that they help poorer households pay for their utility bills by providing a savings mechanism to smooth expenses across months and they make electricity bills less variable and more predictable (Beard et al., 1998; Ha et al., 1993).

Evaluation of bill smoothing programs in the US shows that consumers use more electricity when offered a bill smoothing program (Ha et al., 1993), and that adoption of the program varies substantially with the associated fees (Beard et al., 1998). Because utilities tend to be local monopolies, they can take advantage of a large amount of information on past use of their services. The decentralized nature of the desludging market means that many different service providers have worked with households over time, so no one business will have access to the household's desludging history. While we did not have the data necessary to implement a personalized ‘budget billing’ treatment with monthly bills adapted to the household's past use, our treatment provided households with a nudge to consistently save the amount that they would need for their desludging expenses over time given the median frequency of desludgings in Dakar, the household's subsidy level, and whether they left a pre-paid deposit. While our program served as a nudge toward the average necessary payment level, there was no correction made to the text messages for those who had paid too little or too much in early months.

The three groups received the following monthly text messages.

• Save at will: “Need to empty your latrine pit? Save bit by bit to have XX in the Wari account for each of your two desludgings, then call ZZ. Available until DD/MM/YY.”
• Monthly billing: “Need to empty your latrine pit? Pay YY each month in the Wari account then call ZZ. Your two desludgings are XX each. Available until DD/MM/YY.”
• Pay in full: “Need to empty your latrine pit? Your first two desludgings will cost you XX each, payable by Wari at the time of service. Call ZZ. Available until DD/MM/YY.”

The monetary amounts in the messages varied by treatment, whether the person paid a deposit, and their subsidy level. The pay-in-full treatment was enforced, such that individuals were not able to make deposits any smaller than the full amount, as is common in the general market for desludgings.

In both the save-at-will and monthly-billing treatments individuals were able to make deposits of any size whenever they wanted, so in terms of Wari capabilities they were logistically equivalent. But the monthly-billing messages suggested to participants that deposits should be in the amount requested and advised the individual to “pay.” The save-at-will messages instead advised the individual to “save.”

We give individuals instructions regarding how to withdraw money from their accounts. But, those in the monthly-billing group could potentially have thought that they were paying in advance, rather than saving towards payment, and this could have acted as a deterrent to depositing. Participants in the save-at-will and monthly-billing groups were equally likely to cite lack of understanding as a deterrent to purchasing the subsidized desludging. Themes related to commitment (such as wanting easy access to their money for emergencies) were never cited as deterrents by those who did not use the subsidized desludging when they were asked an open-ended question in the endline.

There were small financial disincentives to making multiple deposits, and these disincentives were relatively larger for small deposits. Clients paid a $0.20 fee to deposit any amount less than $10 and a 2% fee for deposits larger than that. There were no fees for withdrawals or transfers to desludging operators. During the survey, subscribers were told that if they wanted to withdraw money they could call the phone number mentioned in the text messages Monday through Friday from 8 a.m. to 6 p.m. In practice, this only happened once.

Although these accounts may seem like they entail a relatively soft commitment, only through mental accounting, there are some noteworthy features which make the commitment stronger. First, the participants can only withdraw money on weekdays during business hours. Second, even during those hours they have to first make a phone call and then go to a Wari boutique. This involves both a monetary and time cost (making phone calls is not free). They do not have immediate anonymous access. Finally, although participants can withdraw from the account with no fee, there is a fee to deposit in the Wari account. They may hesitate to withdraw money for some purpose other than a desludging since then they would have to pay more fees to deposit more money in order to purchase the desludging. The deposit could also serve as a sunk cost, as people are more likely to use the desludging if they feel that they have already committed money as a partial payment for the service.

People in Dakar often change their phone numbers, so in our endline survey we asked whether the messages we sent were actually received. We find that 68% of respondents say they received messages every month, 14% say they received some messages, and 13% say they did not receive any messages. There are an additional 5% who say they do not know, usually because the person responding to the endline survey was not the same person who responded to the baseline survey and whose phone number was set to receive the text messages. Thus, most people do seem to receive the monthly notifications.

In our original design, we had planned to additionally study the impact of having multiple accounts, one earmarked and one more general. While all subscribers were offered the account earmarked to pay for the mechanized desludging, in a cross-cutting randomization, half of the subscribers were additionally offered a general (not earmarked) account. Of those who had a non-earmarked savings account, 96% of them never touched it, 3% of them deposited into the non-earmarked savings account once, and 1% deposited multiple times. These numbers are not low relative to the literature (Dupas et al., 2018). Given the lack of variation in use of the general savings account, in this paper we do not look at the general account treatment.

It may be surprising that, of the 1496 subscribers, only 19% actually purchased at least one of the two subsidized desludgings. (Only 5% purchased two subsidized mechanized desludgings, and we do not analyze this outcome due to the low variation.) Because our data on purchase of mechanized desludgings through the subscription comes from administrative data, we know this information for all 1496 subscribing households. For the 1380 subscribers who completed our endline survey, we asked them to self-report their use of desludgings over the previous twelve months. Recall appears to be quite accurate; 97.4% of endline respondents who purchased a mechanized desludging through the subscription (as known through our administrative data) reported in the survey that they had purchased a mechanized desludging in the past year. Of endline respondents, 20% purchased a mechanized desludging through the subscription, 21% purchased a mechanized desludging outside of the subscription, 8% hired a non-family member to do a manual desludging, 8% got a family member to do a manual desludging, and 43% did not end up needing any desludging over that time period.

We asked those subscribers who purchased a mechanized desludging outside the subscription why they did not purchase our subsidized service, allowing them to choose as many options as they liked, and the most common (not mutually exclusive) responses were that they did not understand the subscription system (32%), they had trouble making a mobile money deposit (27%),^11^ and they found a better price (23%). Less common explanations were that they lost their card (5%), they forgot (5%), or they wanted to use their usual desludging service provider (4%).

Given that lack of understanding was the most common response, we also look at whether understanding differed across the treatment groups. Lack of understanding was similar in magnitude across the save-at-will and monthly-billing groups. It was given as an explanation for purchasing a mechanized desludging outside of the subscription by 35% and 36% of individuals who purchased an unsubsidized mechanized desludging in those two groups. Understanding appeared to be higher in the pay-in-full group, only being chosen as an explanation by 26% of the individuals who purchased an unsubsidized mechanized desludging in that group, although the difference is not statistically significant.^12^

## 4 Data

We conducted a baseline survey offering the subscription to 3757 households between February and May 2014. Half of the households were offered the high subsidy and 87% were required to leave a deposit in order to sign up. Of those offered the subscription, 1496 signed up. In the original full sample, individuals randomly chosen to have a high subsidy were more likely to sign up for the subscription; 50% of individuals offered the high subsidy subscribed, while only 30% of individuals offered the low subsidy subscribed. Similarly, those with no required deposit were also more likely to sign up; 51% of individuals who were not required to leave a deposit subscribed, while only 38% of individuals who were required to leave a deposit subscribed.

We first explore balance in the full sample in a table shown in the main text, and then explore balance for the mobile money treatments in a table shown in the appendix. We test for balance in the full sample by running regressions of each variable on the subsidy level, the deposit requirement, and grid-point level fixed effects. Table 1 shows that out of 15 variables tested, only two are significantly unbalanced across the groups at the 10% level and none are significantly unbalanced across groups at the 5% level. Thus we do not control for unbalanced covariates in the regressions using the full sample.

**Table 1:** Randomization balance - full sample.

	Mean (SD)	Coefficient (SE)			
	(1)	(2)	(3)	(4)	(5)
	Low Subsidy, No Deposit	High Subsidy (HS)	Deposit Required (DR)	*p*-values HS = DR = 0	Total Obs.
Courtyard looks clean	0.75 (0.43)	0.0061 (0.01)	0.014 (0.02)	0.74	3710
Respondent years of education	6.31 (5.64)	0.100 (0.18)	−0.040 (0.27)	0.85	3746
Respondent has no formal education	0.34 (0.47)	0.0025 (0.02)	−0.0019 (0.02)	0.98	3757
Respondent age	51.0 (14.70)	−0.56 (0.46)	−1.31∗ (0.71)	0.089	3712
Respondent male	0.69 (0.46)	0.025 (0.02)	−0.038 (0.02)	0.082	3756
Household size	10.2 (6.18)	−0.11 (0.19)	−0.051 (0.29)	0.82	3710
Number of rooms in house	6.76 (3.87)	−0.043 (0.12)	−0.12 (0.18)	0.74	3710
Own their house	0.76 (0.43)	0.0057 (0.01)	0.0065 (0.02)	0.88	3710
House has two stories	0.26 (0.44)	0.0058 (0.01)	−0.014 (0.02)	0.74	3757
Wealth index	0.040 (1.74)	−0.00028 (0.05)	0.079 (0.08)	0.59	3710
Respondent has no regular pay	0.77 (0.42)	0.0030 (0.01)	−0.00032 (0.02)	0.98	3757
Used mechanized in year before baseline	0.34 (0.48)	0.0064 (0.01)	0.0044 (0.02)	0.89	3757
Used manual in year before baseline	0.33 (0.47)	0.0035 (0.02)	0.016 (0.02)	0.78	3757
Used mechanized more than a year before baseline	0.12 (0.32)	0.0012 (0.01)	0.020 (0.02)	0.50	3757
Never desludged before baseline	0.28 (0.45)	−0.0027 (0.01)	−0.016 (0.02)	0.77	3757
Responded to endline survey	0.88 (0.32)	0.018∗ (0.01)	0.0057 (0.02)	0.17	3757
*p*-value of joint test		0.744	0.530	0.722	

Note: All variables are measured in the baseline (bl). Column (1) shows the mean and standard deviation of observations with a low subsidy and no deposit requirement. Columns (2) and (3) show the coefficient on high subsidy and deposit requirement in a regression including grid-point level fixed effects. Standard errors are in parentheses: p∗<0.10, p∗∗<0.05, p∗∗∗<0.01 in columns (2) and (3). Column (4) shows the *p*-values for tests of whether the coefficients in columns (2) and (3) equal one another and equal 0. The last row shows the *p*-values for a joint test of all the individual tests in the preceding rows.

The sample of subscribers is split in close to even thirds across the payment treatments (save at will, monthly billing, and pay in full). Table C-1 shows tests for balance in baseline values among subscribers across the three mobile money treatment groups. We run regressions of the variable on the save-at-will treatment, the monthly-billing treatment, and grid-point level fixed effects. The excluded category is the pay-in-full treatment. Out of the 15 non-treatment variables tested, three are unbalanced at the 5% level of significance. The joint test suggests that the save-at-will treatment group is well-balanced, but the monthly-billing group is not. In all regressions studying the effects of the mobile money treatments on the subsample of subscribers we include the unbalanced variables from this table as covariates.

In terms of outcome variables, we have administrative data on mobile money account usage and purchases of the subsidized mechanized desludging subscription for all households. Thus there is no attrition for these outcomes. We conducted an endline survey between March and May 2015 and were able to reach 90% of individuals in the full sample and 92% of subscribers. This endline survey data gives us two additional outcome variables: purchases of unsubsidized mechanized desludgings on the open market, and purchases of any mechanized desludging (either subsidized by us or unsubsidized). The bottom row of Table C-1 shows that attrition is not correlated with the mobile money treatments. The bottom row of Table 1 shows attrition is also not correlated with the deposit requirement. But, individuals who were offered a high subsidy in the baseline are significantly more likely to respond to our endline survey. Because of this, for our first two main tables of results we will additionally calculate Lee (2009) upper and lower bounds on the coefficient on the high subsidy to account for the selective attrition.

Table C-2 shows summary statistics for both the full sample as well as the subsample of subscribers. As mentioned previously, 19% of subscribers did purchase at least one of the subsidized desludgings, while 23% of subscribers deposited money in the account at least once. The average total value deposited in the desludging account (excluding the mandatory initial deposit, and including people who deposited nothing) is around $9. Forty-one percent of subscribers purchase a mechanized desludging between the baseline and the endline.

We call deposits into the earmarked desludging account which did not bring the balance up to the full price of the subsidized desludging ‘non-final deposits.’ Non-final deposits do not include the initial obligatory deposit for those who were required to leave their participation payment as a pre-paid deposit. Non-final deposits were only possible in the save-at-will and monthly-billing treatment groups. We see that 24% of individuals in those treatment groups used the earmarked account at all, and of those 28% made a non-final payment (that is 26% of those in the monthly-billing treatment group and 30% of those in the save-at-will treatment group). For those who made such deposits, the modal number of non-final payments was 1, the average number was 2, and the average total value was $19. We hypothesize that the relatively low use of non-final deposits is either due to the fact that people did not have experience saving in Wari accounts in the past or that they were not able to or did not want to save.^13^

From administrative data we also know the timing of all deposits and purchased subsidized desludgings. On average, conditional on making a deposit, households waited 111 days after their interview before making their first deposit. Those who purchased a subsidized desludging waited an average of 14 days (with a standard deviation of 49 days) between making their first deposit and purchasing the desludging. Given that without our intervention individuals must pay for the service in full at the time of purchase, this means that our intervention allowed individuals to start saving in advance at a moment when they more conveniently had cash on hand. Individuals also waited an average of 3 days between making their last deposit and purchasing the desludging. Individuals in all three treatment arms could wait as long as they liked between when their account reached the balance necessary to purchase a desludging, and when they actually purchased it, potentially using the accounts as a safe place to keep earmarked funds. (Of course the pay-in-full group was required to deposit the full amount at one time, while the other two groups could deposit little by little if they so desired.)

As discussed in more detail in Section 5, we analyze heterogeneous impacts based on two dimensions: the household's previous mechanized desludging history, and their access to a job with a regular salary. The summary statistics show that in the full sample, 29% of households have used a mechanized desludging in the year before the baseline survey; 13% have used a mechanized desludging in the past but this desludging took place more than a year before the baseline; 31% have purchased a manual desludging at any point in the past but never a mechanized desludging; and 27% have never desludged their current pit. We also find that 21% of respondents have a salaried job, such as a job as a civil servant, in the armed forces, or as a private sector employee with a monthly salary. The summary statistics are similar for the sub-sample of subscribers, though subscribers are more likely to have used a mechanized desludging in the past and more likely to have jobs with a regular salary.

## 5 Estimation strategy

We estimate the impacts of the subsidy level and the deposit requirement on the full sample and the impacts of the three mobile money payment options on the subsample of subscribers. For the full sample our four outcome variables are whether they signed up (subscribed), purchased a subsidized mechanized desludging through the research project, purchased a mechanized desludging on the open market, or purchased any mechanized desludging at all. For the sample of subscribers we look at three of the same outcomes (excluding whether they signed up, since all subscribers by definition signed up) and in addition we look at whether they made any deposits or any non-final deposits and the total value of deposits and non-final deposits. Our regression equation is:

$$y_{ig}=\alpha +t_{ig}^{\prime }\beta +x_{ig}^{\prime }\gamma +\psi_{g}+\varepsilon_{ig}$$

where *y* is the outcome of interest for household *i* which was selected using starting grid-point *g*, *t* is a vector of treatment dummies, *x* is a vector of individual and household characteristics, and *ψ*_*g*_ are grid-point fixed effects.

Because our main outcome of interest is desludging use in the year after the baseline, in the covariate vector *x* we control for mechanized and manual desludging use in the year before the baseline. Controlling for past values of outcome variables increases precision relative to differences-in-differences specifications, particularly in cases where the baseline and endline measures are not measured in exactly the same way and in which the autocorrelation between outcomes is low (McKenzie, 2012). We also include fixed effects at the grid-point level because, as discussed in Section 3, the subsidy and deposit randomization were stratified at that level. Finally, in the subscriber regressions we include controls for baseline characteristics that were significantly unbalanced at the 5% level across the mobile money treatment groups: household wealth and household size.

For the sub-sample of subscribers who made deposits, we also run a similar regression where the outcome variable is the number of days between different events such as the survey interview, the first deposit, the last deposit, and the purchase of the subsidized desludging. Also for the sub-sample of subscribers (whether or not they made a deposit) we look at the impact of the timing of the text-message reminder on the timing of deposits. For each household we break up their subscription year into three-day periods starting with the day of their first reminder. So, the first three-day period is the day the reminder comes and the two following days. The next three-day period is three to five days after the first reminder. The three-day periods are different for each household since they are interviewed and sign up at different times and thus receive their reminders on different dates. This allows us to control for day of week (e.g., Monday), calendar month (e.g., January), calendar day (e.g., the first of the month), and reminder number fixed effects. Our outcome variable of interest is *y*_*dig*_ which measures whether household *i* made a deposit in three-day period *d*. The control vector *m*_*dig*_ includes controls such as whether the period immediately follows the text message, whether it is three to five days after the text message, etc. We also interact the treatment variables with *m*_*dig*_ to test whether the text messages are more salient and effective for people in the save-at-will group. For this regression we cluster our standard errors at the household level. The basic equation we estimate is:

$$y_{\mathrm{dig}}=\alpha +t_{ig}^{\prime }\beta +x_{ig}^{\prime }\gamma +m_{\mathrm{dig}}\rho +m_{\mathrm{dig}}t_{ig}^{\prime }\phi +\psi_{g}+\varepsilon_{\mathrm{dig}}$$

Going back to the original regressions, we also look for heterogeneous impacts on two dimensions: regularity of income and mechanized desludging history. In this case we use the following set of regressions:

$$y_{ig}=\alpha +t_{ig}^{\prime }\beta +x_{ig}^{\prime }\gamma +h_{ig}\rho +h_{ig}t_{ig}^{\prime }\phi +\psi_{g}+\varepsilon_{ig}$$

where *h*_*ig*_ represents the trait for which we are measuring heterogeneous impacts. The effect of the mobile money treatment group on the subset of people with a specific character trait equals *β* + *ϕ*.

The first dimension of heterogeneity we look at, whether the individual who made the original decision to sign up for the subscription and who has the Wari account, has a job with a regular monthly salary, is suggested by Dupas et al. (2018). They focus their savings interventions on individuals who do not have regular wages under the assumption that these are the individuals who most urgently need access to savings accounts. Individuals with regular wage-paying jobs may benefit from the mobile money interventions less since they do not experience as much variation in their income as those who are self-employed or have more transitory jobs.

The second dimension of heterogeneity we look at is previous mechanized desludging history. People who have purchased a more sanitary mechanized desludging in the past year are more likely to continue purchasing mechanized desludgings in the future and so, while they may appreciate the option to save at will, our interventions are unlikely to change their general purchases of mechanized versus manual desludgings. On the other hand, people who have purchased a mechanized desludging in the past, but over a year ago, are more likely to be individuals who are on the fence between the two mechanisms and for whom our treatments might have an impact on the type of desludging they choose. People who have never had a desludging in the past year, or who have only had manual desludgings, are likely to be the least affected by our interventions.

## 6 Results

We will first look at the effects of the different subsidy levels and deposit requirements on outcomes in the full sample, and then look at the impact of the mobile money treatments which were randomized over the subset which signed up for the subsidized desludging subscription. In Table 2 we examine the influence of the randomized subsidy and deposit treatments on take-up, thereby determining our sample of subscribers. We also look at how these treatments influenced desludging behavior. Column (1) shows that receiving the larger subsidy increases the likelihood that an individual signs up by 20 percentage points. This then translates into an eight percentage point increased likelihood of purchasing a subsidized desludging through our program, a six percentage point lower likelihood of purchasing a subsidized desludging on the open market, and a three percentage point increase in the likelihood of purchasing a mechanized desludging overall. The large and significant coefficients on having a high subsidy show, as have many others before us (Bates et al., 2012), that subsidies on preventive health products increase their take-up.

**Table 2:** Average impact of subsidy and deposit requirement on full sample.

	(1)	(2)	(3)	(4)
	Signed Up	Subsidized Desludging	Unsubsidized Mechanized Desludging	Any Mechanized Desludging
High subsidy	0.203∗∗∗ (0.016)	0.084∗∗∗ (0.009)	−0.059∗∗∗ (0.014)	0.026∗ (0.014)
Deposit required	−0.104∗∗∗ (0.024)	−0.008 (0.013)	0.008 (0.021)	−0.006 (0.021)
Mechanized desludging in year before baseline	0.112∗∗∗ (0.019)	0.074∗∗∗ (0.011)	0.382∗∗∗ (0.017)	0.464∗∗∗ (0.017)
Manual desludging in year before baseline	−0.004 (0.017)	0.016∗ (0.010)	0.000 (0.015)	0.021 (0.015)
*R*^2^	0.245	0.179	0.339	0.402
Mean of Dependent Variable	0.398	0.074	0.237	0.315
*N*	3757	3757	3395	3395

Note: All regressions include fixed effects at the grid-point level. Standard errors are in parentheses: p∗<0.10, p∗∗<0.05, p∗∗∗<0.01. Outcome variables for the full sample are (1) signed up for the subsidized mechanized desludging through the subscription, (2) purchased the subsidized mechanized desludging through the subscription, (3) purchased an unsubsidized mechanized desludging between the baseline and endline, and (4) purchased any mechanized desludging between the baseline and the endline. Lee (2009) lower and upper bounds (and their standard errors) accounting for selective attrition in the endline survey for the coefficient on high subsidy in column (3) are −0.059∗∗∗ (0.017) and −0.040∗∗∗ (0.015) and in column (4) are 0.026 (0.018) and 0.045∗∗∗ (0.016).

Because Table 1 showed that individuals with the high subsidy were more likely to respond to the endline survey, we also calculate Lee (2009) upper and lower bounds for the two outcomes which come from the endline survey: purchasing an unsubsidized mechanized desludging and purchasing any mechanized desludging. This procedure gives an upper and lower bound on the coefficient in a regression with no other controls under the monotonicity assumption that treatment affects attrition in only one direction (e.g., that receiving a high subsidy makes people more likely to respond to the endline survey, which seems plausible in our context). The results are shown in the note under Table 2. The magnitudes of the bounds are in a similar range as the coefficients in the OLS regression and three out of four of the bounds are statistically significantly different from zero.

Having to leave a deposit does discourage people from signing up, but it appears to mostly discourage those who were not actually going to purchase the subsidized desludging anyway, since it has no impact on purchases. At least in this setting, requiring a deposit appears to be a useful way of deterring cheap talk and gauging interest, without having a detrimental effect on final usage. In terms of external validity, we did pay individuals to participate in our survey and they could either use that money as their deposit or keep it. In settings where the program does not give individuals the funding necessary for the deposit, the results might be different.

**Fig. 1:**
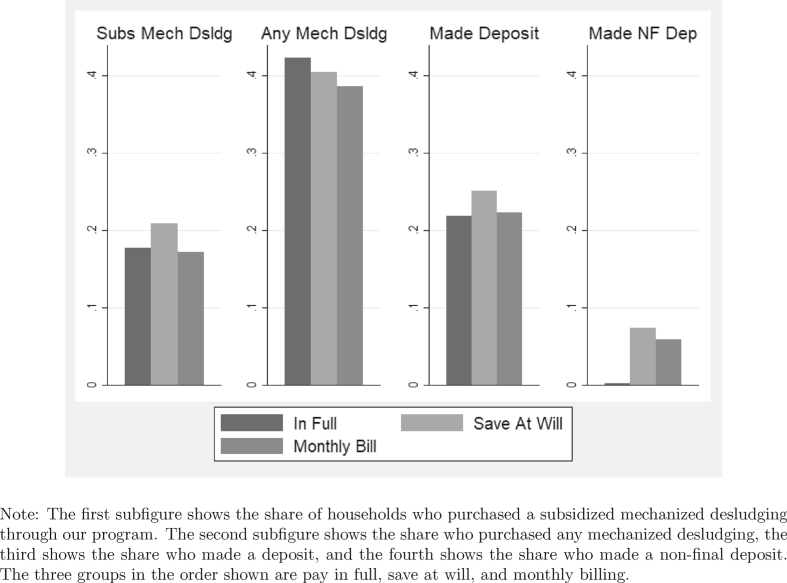
Average desludging and account use by treatment group.

Next we look at the effect of the mobile money treatments on the subscribers. Before conducting a regression analysis, we first look visually at purchases of mechanized desludgings and usage of the account in Fig. 1. In the first panel we can see that the save-at-will group purchases the subsidized desludging at a higher rate than either the pay-in-full or monthly-billing treatments. In the third panel we see that they use the account more than those in the other two groups. The fourth panel shows evidence of a likely mechanism: individuals in the save-at-will group are more likely to make non-final deposits than those in the monthly-billing group.^14^ Still, the second panel shows no clear impact on overall purchases of mechanized desludgings (combining subsidized mechanized desludgings through our project and mechanized desludgings purchased on the open market).

In Table 3 we show the same results, but in a regression analysis. In columns (1) through (3) we study the impact of the mobile money treatments on purchases of mechanized desludgings both from the research project and on the general market. Column (1) shows that the save-at-will treatment increases the probability of purchasing the subsidized mechanized desludging by five percentage points compared to being in the treatment group which was forced to pay in full. This is a 26% increase in purchases of subsidized desludgings at the mean.

**Table 3:** Average impact of mobile money treatments on subscribers.

	(1)	(2)	(3)	(4)	(5)	(6)	(7)
	Subsidized Desludging	Unsubsidized Mechanized Desludging	Any Mechanized Desludging	Any Deposits	Value of Deposits	Any Non-Final Deposits	Value of Non-Final Deposits
Save at will	0.049∗ (0.028)	−0.076∗∗∗ (0.029)	−0.015 (0.033)	0.051∗ (0.030)	0.838 (0.720)	0.027 (0.020)	0.483∗ (0.264)
Monthly billing	−0.008 (0.029)	−0.029 (0.030)	−0.033 (0.034)	0.008 (0.031)	0.072 (0.740)		
High subsidy	0.123∗∗∗ (0.024)	−0.080∗∗∗ (0.025)	0.036 (0.028)	0.123∗∗∗ (0.026)	1.778∗∗∗ (0.612)	0.016 (0.020)	0.216 (0.262)
Deposit required	0.023 (0.031)	0.014 (0.032)	0.041 (0.037)	0.014 (0.034)	0.061 (0.807)	−0.043 (0.028)	−0.694∗ (0.355)
Mechanized desludging in year before baseline	0.097∗∗∗ (0.026)	0.275∗∗∗ (0.028)	0.386∗∗∗ (0.031)	0.102∗∗∗ (0.029)	3.032∗∗∗ (0.684)	−0.031 (0.023)	−0.134 (0.302)
Manual desludging in year before baseline	0.047∗ (0.026)	0.002 (0.028)	0.068∗∗ (0.031)	0.017 (0.029)	0.504 (0.681)	−0.044∗ (0.023)	−0.436 (0.298)
Household size	0.002 (0.002)	0.001 (0.002)	0.004∗ (0.003)	0.002 (0.002)	0.123∗∗ (0.057)	0.002 (0.002)	0.013 (0.025)
Wealth index	0.020∗∗ (0.008)	0.004 (0.008)	0.026∗∗∗ (0.009)	0.024∗∗∗ (0.009)	0.581∗∗∗ (0.203)	0.014∗∗ (0.007)	0.252∗∗∗ (0.086)
*R*^2^	0.321	0.403	0.467	0.308	0.289	0.367	0.304
Mean of Dependent Variable	0.188	0.212	0.406	0.233	4.723	0.067	0.652
*N*	1479	1365	1365	1479	1479	998	998

Note: All regressions include fixed effects at the grid-point level. Standard errors are in parentheses: p∗<0.10, p∗∗<0.05, p∗∗∗<0.01. Outcome variables are (1) purchased the subsidized mechanized desludging through the subscription, (2) purchased an unsubsidized mechanized desludging between the baseline and endline, (3) purchased any mechanized desludging between the baseline and endline, (4) used the desludging account, and (5) total value deposited in the desludging account (in thousands of CFA, 1000 CFA is approximately $2). Outcome variables for the save-at-will and monthly-billing groups only are (6) number of non-final deposits and (7) value of non-final deposits (in thousands of CFA). In columns (1) through (5) the excluded treatment group control variable is pay in full. In columns (6) and (7) we drop the pay-in-full group, and the excluded treatment group control variable is monthly billing. Lee lower and upper bounds (and their standard errors) accounting for selective attrition in the endline survey for the coefficient on high subsidy in column (2) are −0.073∗∗∗ (0.027) and −0.050∗∗ (0.023) and in column (3) are 0.049∗ (0.029) and 0.073∗∗ (0.028).

Does the increase in purchases of subsidized mechanized desludgings in the save-at-will group imply an increase overall in the purchase of mechanized desludgings? Asked differently, does the save-at-will treatment induce individuals to switch from purchasing unsubsidized *mechanized* to subsidized mechanized desludgings, or does it induce households to switch from purchasing unsubsidized *manual* to subsidized mechanized desludgings? Because of the large negative externalities associated with manual desludging, we are primarily interested in increasing the overall purchase of mechanized desludging rather than taking business away from the existing mechanized desludging market. To look at these outcomes in columns (2) and (3), we must use self-reported data rather than using administrative data and we only have this data for those individuals who completed the endline survey.

The mobile money treatments do not seem to induce individuals to switch from manual to mechanized desludgings. Column (2) shows that the save-at-will treatment causes a decrease in the purchase of unsubsidized mechanized desludgings outside of our system. This effect is of opposite sign but similar in magnitude to the increase in purchases of subsidized mechanized desludgings. When we look at the purchases of mechanized desludgings more generally in column (3), we see that the impact of the save-at-will option on purchases of mechanized desludgings overall is small and insignificant. This suggests that the save-at-will treatment merely induces individuals who were going to purchase a mechanized desludging anyway to purchase one through our system rather than on the open market. The earmarked savings account and mental accounting nudge does not increase mechanized desludging overall, and so likely does not improve sanitation.

Note that in column (3) looking at the purchase of any mechanized desludging, the coefficient on the high subsidy is a positive but insignificant 0.036. This is a bit larger but similar in magnitude to the coefficient of 0.026 found in Table 2. The note below the table shows that the Lee (2009) bounds on the coefficient (for a regression on the sample of subscribers with no other controls) are 0.049 and 0.073, both statistically significant. We should not interpret the insignificant subsidy coefficient in Table 3 as implying that the high subsidy does not affect overall purchases of mechanized desludgings. This table explores the effects of the mobile money treatments on subscribers only since this is the group that was assigned a mobile money treatment group. This select group contains a higher share of individuals assigned to a high subsidy and no deposit than the full sample. Because the high subsidy significantly increases the likelihood of signing up (column (1) of Table 2) and then, conditional on signing up, increases the likelihood of purchasing a mechanized desludging (positive though insignificant coefficient in column (3) of Table 3), the coefficient on the subsidy on overall purchases in the full sample is positive and significant (column (4) of Table 2). Thus our interpretation is that the high subsidy did encourage individuals to switch from manual to mechanized desludgings, while the mobile money savings treatments did not.

While we are unable to clearly identify why the mental accounting savings program did not cause households to switch from using a manual desludging to a mechanized desludging, one potential explanation is that households unsure of whether they would be able to save enough for a mechanized desludging were unwilling to commit funds to an earmarked account. This would be in line with the results found in Dupas and Robinson (2013). Note also that because the mobile money treatments were only offered to those who signed up for the subsidized mechanized desludging, many individuals who might have been convinced by new mobile money savings options to switch from manual to mechanized may have already been screened out of the market when they were first asked whether they wanted to subscribe.

In columns (4) through (7) of Table 3 we look at individuals’ deposit behavior. While many of the coefficients are weak in terms of statistical significance, the magnitudes are relatively large. We first look at deposits of any sort. Individuals in the save-at-will group are five percentage points more likely to make a deposit than are those in the pay-in-full group.

Why does the save-at-will treatment increase purchase of the subsidized mechanized desludging? One reason might be because it allows individuals to make non-final deposits when they have cash on hand. Non-final deposits are those which do not result in the account containing at least the amount necessary for the individual to purchase a subsidized mechanized desludging.

Columns (6) and (7) look at whether households in the save-at-will treatment were more likely to make non-final deposits or saved a higher value of non-final deposits compared to the monthly-billing group. Individuals in the save-at-will group deposit almost $1 more (483 CFA) in the form of non-final deposits than do individuals in the monthly-billing group. While this result is only significant at the 10% level, it gives suggestive evidence that the flexibility afforded by the save-at-will treatment encourages people to make non-final deposits and then makes it more likely that they purchase the subsidized desludging.

Note that households in the pay-in-full treatment group are dropped in these two final columns since they were not allowed to make non-final deposits. Thus, the results only compare households in the save-at-will treatment to those in the monthly-billing treatment group. If we were instead to leave all three treatment groups in the regression and compare non-final deposits of those in the save-at-will treatment to those in the omitted category of pay in full, we would see that households in the save-at-will treatment are seven percentage points more likely to make non-final deposits than the pay-in-full group (*p*-value of 0.000) and make non-final deposits approximately $1.50 (787 CFA) higher than those in the pay-in-full group (*p*-value of 0.000). The fourth panel of Fig. 1 also gives a clear representation of this relationship.

In sum, the save-at-will treatment appears to encourage individuals who were going to purchase a mechanized desludging on the open market, to instead purchase their mechanized desludging through our subsidized subscription program. Part of the mechanism behind this effect appears to be the flexibility offered by the fact that individuals could make non-final deposits of any size and at any time into these accounts.

In contrast to the effects of the save-at-will treatment, columns (1) to (5) of Table 3 show no impact of the monthly-billing treatment. The purchases of mechanized desludgings and overall deposit behavior are no different for those in the monthly-billing and pay-in-full groups. In the next subsection we will look more closely at both the timing and size of deposits across the different treatment groups to better understand the mechanism behind these results.

### 6.1 Timing and size of deposits

In this subsection we look at the timing of deposits both with respect to the timing of the desludging (i.e., how far in advance households begin anticipatory saving) and with respect to the timing of the reminder text messages (i.e., how many days after receiving a reminder households make deposits). We also look at how the size of the deposit made compares with the deposit size suggested by the text message in the monthly-billing treatment.

In the previous section, we saw that the save-at-will treatment makes it more likely for households to purchase the desludging through the subscription. The suggested mechanism is that it gives them a way to save money whenever they have extra on hand. This was evidenced by the fact that individuals in the save-at-will group are more likely to make non-final deposits, and also make larger non-final deposits. There was no such effect of the monthly-billing treatment.

If the save-at-will treatment helps individuals to save over time and thereby facilitates their purchase of a subsidized desludging, we should also see an effect of the treatment on the timing of deposits and purchases. The option to save over time could decrease the time between interview and first deposit (decreasing the time it takes individuals to start saving) or increase the time between first deposit and desludging (increasing the amount of time the money is saved in anticipation in the mobile money account).

Because we can only study the timing of deposits and purchases for those who actually deposit and purchase, the results suffer from sample selection and thus are only descriptive. Still, the results provide suggestive evidence on the extent to which households are taking advantage of the accounts to save anticipatorily before they purchase their desludging.

We look at the average treatment effects on timing in Table 4. Given that households usually wait until they need a desludging to purchase one, and once they need one they do not have much flexibility to wait, we do not expect to see any effect of the treatments on the days between the interview and the desludging. This is confirmed in column (1). Neither do we see large significant differences between the groups in terms of the time from the interview to the first deposit (column (2)) or last deposit to first desludging (column (4)). In contrast, households in the save-at-will group wait on average 47% more days (approximately 7 days longer) between their first deposit and their desludging (column (3)). There is no significant effect of the monthly-billing treatment. This suggests that a benefit of the save-at-will treatment is that it allows subscribers to engage in anticipatory savings, putting money away in advance of when they need to purchase a desludging.

**Table 4:** Timing of deposits and desludging: outcome is ‘Log of days from … ’.

	(1)	(2)	(3)	(4)
	Interview to First Desludging	Interview to First Deposit	First Deposit to First Desludging	Last Deposit to First Desludging
Save at will	0.222 (0.153)	−0.019 (0.156)	0.473∗∗ (0.202)	−0.082 (0.127)
Monthly billing	0.254 (0.160)	−0.084 (0.160)	0.216 (0.211)	−0.218 (0.133)
High subsidy	−0.197 (0.153)	0.033 (0.148)	−0.493∗∗ (0.202)	−0.071 (0.127)
Deposit required	−0.195 (0.183)	0.065 (0.181)	−0.583∗∗ (0.241)	−0.323∗∗ (0.152)
*R*^2^	0.114	0.023	0.090	0.070
Mean of Dependent Variable in Logs	4.434	4.221	0.913	0.572
Mean of Dependent Variable in Levels	128	111	14	3
*N*	278	345	278	278

Note: Controls in all regressions include: pre-intervention outcomes (mechanized desludging in year before baseline and manual desludging in year before baseline) and unbalanced controls (hhd wealth and hhd size). Standard errors are in parentheses: p∗<0.10, p∗∗<0.05, p∗∗∗<0.01. Outcomes are (1) log of days from interview to first desludging, (2) log of days from interview to first deposit, (3) log of days from first deposit to first desludging, and (4) log of days from last deposit to first desludging. The excluded treatment group control variable is pay in full.

In sum, the increased purchase of subsidized desludgings evidenced in the save-at-will group is accompanied by an increase in the value of non-final deposits, and an increase in the amount of time which users save for the desludging they anticipate needing in the future. Putting these results together suggests a mechanism for the relative popularity of the save-at-will treatment.

In order to explore whether the monthly text message reminders contribute to the large impact of the save-at-will treatment on purchases of the subsidized desludging, we look at whether individuals are more likely to make deposits in the days after receiving the text message compared to other days of the month, and whether this effect is larger for those in the save-at-will treatment group. All individuals received monthly reminders on different dates, with the first reminder arriving two weeks after their interview, new reminders sent monthly after that a total of 12 times, and then a final reminder one week before the account was closed (which was one week after the 12th reminder). Because individuals in all groups received text messages, we can not measure the impact of receiving a text message compared to not receiving one. But, the text message which was sent to the save-at-will treatment group might make more salient that the purpose of the account was to save for mechanized desludgings and so we might expect to see a greater impact of the reminder in that treatment group.

Table 5 shows that deposits are 34% more likely in the nine days after receiving a text message (excluding the final two weeks of the program) than they are other days of the month. The probability of a deposit being made in any 3-day period is 0.27%, and deposits are 0.091 percentage points more likely to be made in the nine days after receiving a message. Individuals in the save-at-will group are more likely to make a deposit at any point in time, but there is no differential effect of the save-at-will or monthly-billing treatments on the impact of the timing of the text messages. The results on timing of deposits are in accord with the mechanism that the save-at-will treatment increased purchases by giving individuals the opportunity to save when they had extra money on hand, which was uncorrelated with the timing of the reminder text message.

**Table 5:** Impact of reminder on timing of deposits.

	Made at Least One Deposit in 3-day Period × 100							
	(1)	(2)	(3)	(4)	(5)	(6)	(7)	(8)
Reminder day plus 0–2	0.031 (0.059)	0.026 (0.059)	0.027 (0.059)					
Reminder day plus 3–5	0.117∗ (0.064)	0.113∗ (0.064)	0.124∗ (0.065)					
Reminder day plus 6–8	0.098 (0.063)	0.094 (0.063)	0.102 (0.064)					
Reminder day plus 0-8				0.091∗∗∗ (0.033)	0.100∗∗ (0.046)	0.096∗∗ (0.046)	0.100∗∗ (0.046)	0.193∗∗ (0.093)
Reminder day plus 9–11	−0.111∗∗ (0.050)	−0.112∗∗ (0.050)	−0.117∗∗ (0.050)					
Reminder day plus 12–14	−0.049 (0.053)	−0.051 (0.053)	−0.050 (0.053)					
Reminder day plus 15–17	0.043 (0.056)	0.044 (0.056)	0.049 (0.056)					
Reminder day plus 18–20	0.037 (0.063)	0.037 (0.063)	0.046 (0.063)					
Reminder day plus 21–23	−0.037 (0.054)	−0.036 (0.054)	−0.031 (0.055)					
Reminder day plus 24–26	0.049 (0.060)	0.050 (0.060)	0.053 (0.060)					
Save at will	0.137∗∗∗ (0.041)	0.137∗∗∗ (0.041)	0.137∗∗∗ (0.041)	0.137∗∗∗ (0.041)	0.137∗∗∗ (0.042)	0.137∗∗∗ (0.042)	0.139∗∗∗ (0.042)	0.140∗∗∗ (0.042)
Monthly billing	0.055 (0.037)	0.055 (0.037)	0.055 (0.037)	0.055 (0.037)	0.062 (0.042)	0.062 (0.042)	0.061 (0.042)	0.062 (0.042)
Deposit required	−0.024 (0.052)	−0.024 (0.052)	−0.022 (0.052)	−0.022 (0.052)	−0.024 (0.052)	−0.024 (0.052)	−0.022 (0.052)	0.011 (0.051)
High subsidy	0.161∗∗∗ (0.036)	0.161∗∗∗ (0.036)	0.163∗∗∗ (0.036)	0.163∗∗∗ (0.036)	0.161∗∗∗ (0.036)	0.161∗∗∗ (0.036)	0.163∗∗∗ (0.036)	0.162∗∗∗ (0.038)
Reminder day plus 0–8 × Save at will					−0.001 (0.074)	−0.000 (0.074)	−0.007 (0.074)	−0.010 (0.073)
Reminder day plus 0–8 × Monthly billing					−0.023 (0.078)	−0.024 (0.078)	−0.020 (0.078)	−0.023 (0.078)
Reminder day plus 0–8 × Deposit required								−0.110 (0.085)
Reminder day plus 0–8 × High subsidy								0.003 (0.062)
Grid-point FE	Yes	Yes	Yes	Yes	Yes	Yes	Yes	Yes
Calendar month FE	No	Yes	Yes	Yes	No	Yes	Yes	Yes
Day of week FE	No	Yes	Yes	Yes	No	Yes	Yes	Yes
Calendar day FE	No	No	Yes	Yes	No	No	Yes	Yes
Reminder # FE	No	No	Yes	Yes	No	No	Yes	Yes
*R*^2^	0.004	0.005	0.006	0.005	0.004	0.005	0.005	0.005
*N*	162,690	162,690	162,690	162,690	162,690	162,690	162,690	162,690

Note: Controls in all regressions include: pre-intervention outcomes (mechanized desludging in year before baseline and manual desludging in year before baseline) and unbalanced controls (hhd wealth and hhd size). Standard errors clustered at the household level are in parentheses: p∗<0.10, p∗∗<0.05, p∗∗∗<0.01. Each observations is a three-day period for a household. The outcome is whether the household deposited in that period (scaled up by 100). The mean of the outcome variable is 0.27.

The results thus far do not hint at why it might be that the monthly-billing treatment did not increase take up of the subsidized desludgings. One might worry that monthly-billing subjects were discouraged by the text messages they received if the suggested amount was too high. But, if anything, non-final deposits are higher in the save-at-will treatment than they are in the monthly-billing treatment suggesting that, when given flexibility, individuals actually wanted to deposit larger amounts rather than smaller amounts.

The monthly deposit schedule may have failed because, given the fee structure of Wari deposits, it is more costly for clients to make the small monthly deposits encouraged by the monthly-billing messages than to make periodic larger payments. Clients paid a $0.20 fee to deposit any amount less than $10 and a 2% fee for deposits larger than $10. But, depending on the amount of the subsidy and whether the household had left a deposit, monthly-billing messages requested people pay between $5.20 and $10.80 each month, which would lead to more fees than if they had made fewer larger payments.

We know the suggested deposit amount for people in the monthly-billing group, and we can calculate what the suggested amount would have been for people in the save-at-will group. Individuals in the monthly-billing group are more likely to make non-final deposits equal to the amount suggested in the text message. Of non-final deposits made in the monthly-billing group, 23% are equal to the amount suggested in the text message, while only 9% of non-final deposits made by individuals in the save-at-will group equal the suggested amount.

Of course we would expect it to be rather rare that individuals in the save-at-will group would happen by chance to make a deposit with the exact value that would have been suggested to them. What may be more interesting is how often they make deposits which are larger than the suggested amount. The monthly-billing treatment may have been less effective because it made people feel like there was a limit on the amount they ought to deposit; it is not very common to over-pay a bill. We find that 70% of non-final deposits made by individuals in the save-at-will group are above what their suggested amount would have been, whereas only 36% of non-final deposits made by individuals in the monthly-billing group are above the suggested amount they received in their text message bill.

**Fig. 2:**
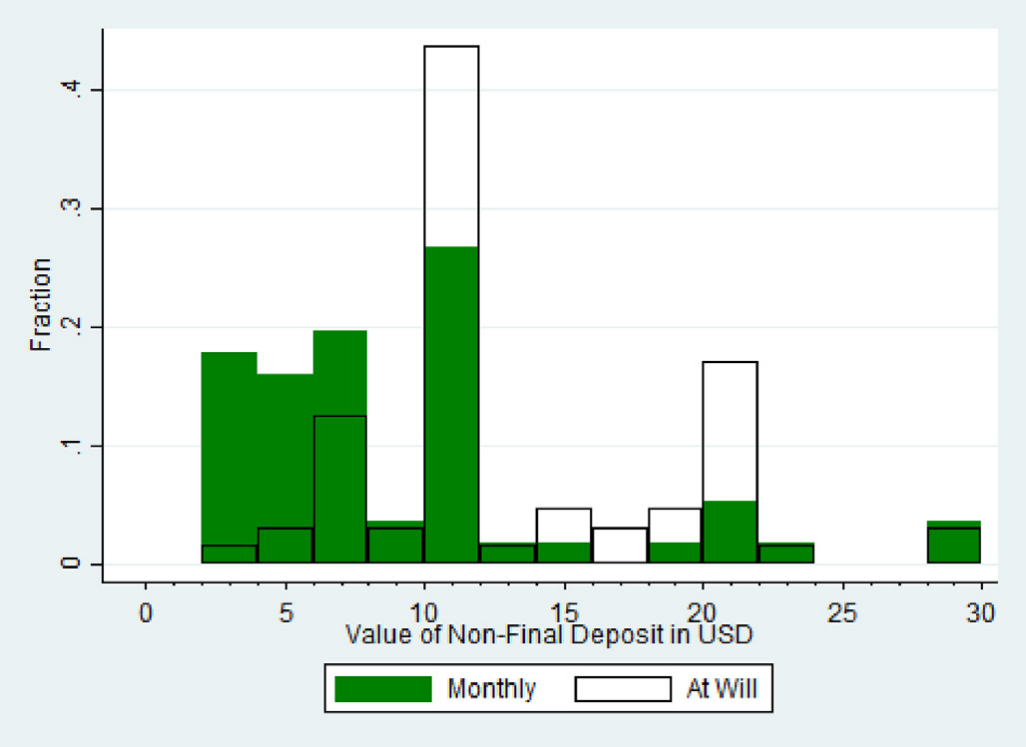
Value of non-final deposits.

Our results are similar in spirt to findings of Thaler and Sunstein (2008) and Stewart (2009) that when credit cards give information on minimum repayment amounts, information which was intended to help consumers, these nudges in fact lead them to repay less and incur higher interest. Fig. 2 shows the distribution of non-final deposits across the two groups which were allowed to make them. Only 19% of non-final deposits made in the save-at-will treatment group were under $10, the cutoff below which the fee was greater than 2%. Of those individuals in the save-at-will group who made at least one non-final deposit, only 19% made at least one deposit that was under $10. On the other hand, 57% of non-final deposits in the monthly-billing group were under the $10 threshold at which the cost structure of deposits changed. Half of the individuals in that group who made non-final deposits made at least one deposit that was under $10. In the end, the intervention may have inadvertently encouraged the monthly-billing households to make frequent, more costly deposits. Ex ante we believed that the individuals in the monthly-billing group might have benefited from the consistency of paying a specified amount each month, but no such benefit was found in practice.

### 6.2 Heterogeneous effects

In this section we look at heterogeneous effects on two dimensions: job stability and history of mechanized desludgings. In Table 6, Table 7 we look for heterogeneous impacts with respect to whether the decider has a job with a regular monthly salary (for example a civil servant or a private sector employee who is paid monthly). To the extent that the mobile money accounts are particularly useful for the households that are unable to save on their own, we may expect households in which the decision maker does not have a regular monthly salary to be more affected by the ability to save at will. This was suggested by Dupas et al. (2018) who specifically focus savings interventions on the population of individuals who do not have regular wage-paying jobs.

**Table 6:** Heterogeneous impacts in the full sample: decider has no regular pay.

	(1)	(2)	(3)	(4)
	Signed Up	Subsidized Desludging	Unsubsidized Mechanized Desludging	Any Mechanized Desludging
High subsidy	0.245∗∗∗ (0.034)	0.140∗∗∗ (0.019)	−0.087∗∗∗ (0.030)	0.061∗∗ (0.031)
Deposit required	−0.076 (0.051)	−0.042 (0.028)	0.003 (0.044)	−0.052 (0.046)
No regular pay	−0.039 (0.057)	−0.035 (0.032)	−0.004 (0.050)	−0.046 (0.052)
High subsidy × No regular pay	−0.053 (0.038)	−0.071∗∗∗ (0.021)	0.035 (0.033)	−0.044 (0.035)
Deposit required × No regular pay	−0.036 (0.057)	0.042 (0.032)	0.008 (0.050)	0.058 (0.052)
*R*^2^	0.251	0.185	0.340	0.403
Mean of Dependent Variable	0.398	0.074	0.237	0.315
*N*	3757	3757	3395	3395
Combined Effects:				
High subsidy if no regular pay	0.192∗∗∗ (0.017)	0.069∗∗∗ (0.010)	−0.052∗∗∗ (0.015)	0.017 (0.016)
Deposit if no regular pay	−0.112∗∗∗ (0.027)	0.001 (0.015)	0.010 (0.023)	0.006 (0.024)

Note: Controls in all regressions include: pre-intervention outcomes (mechanized desludging in year before baseline and manual desludging in year before baseline) and fixed effects at the grid-point level. Standard errors are in parentheses: p∗<0.10, p∗∗<0.05, p∗∗∗<0.01. Having regular employment is measured in the baseline. Outcome variables are (1) signed up for the subsidized mechanized desludging through the subscription, (2) purchased the subsidized mechanized desludging through the subscription, (3) purchased an unsubsidized mechanized desludging between the baseline and endline, and (4) purchased any mechanized desludging between the baseline and the endline. In the lower panel, ‘High subsidy if no regular pay’ shows the value of the sum of the coefficient on ‘High subsidy’ and ‘High subsidy x No regular pay,’ the heterogeneous treatment effect.

**Table 7:** Heterogeneous impacts in the subscriber sample: decider has no regular pay.

	(1)	(2)	(3)	(4)	(5)	(6)	(7)
	Subsidized Desludging	Unsubsidized Mechanized Desludging	Mechanized Desludging	Any Deposits	Value of Deposits	Any Non-Final Deposits	Value of Non-Final Deposits
Save at will	−0.005 (0.055)	−0.103∗ (0.058)	−0.119∗ (0.066)	−0.049 (0.060)	−0.888 (1.420)	−0.007 (0.038)	0.457 (0.489)
Monthly billing	0.029 (0.053)	−0.129∗∗ (0.056)	−0.088 (0.063)	0.016 (0.058)	0.437 (1.374)		
No regular pay	−0.051 (0.046)	−0.038 (0.048)	−0.110∗∗ (0.055)	−0.103∗∗ (0.050)	−1.696 (1.186)	−0.024 (0.032)	−0.068 (0.417)
Save at will × No regular pay	0.074 (0.064)	0.036 (0.067)	0.141∗ (0.076)	0.136∗ (0.070)	2.358 (1.658)	0.048 (0.045)	0.040 (0.578)
Monthly billing × No regular pay	−0.055 (0.063)	0.141∗∗ (0.066)	0.077 (0.075)	−0.014 (0.069)	−0.565 (1.632)		
*R*^2^	0.325	0.407	0.470	0.315	0.293	0.368	0.304
Mean of Dependent Variable	0.188	0.212	0.406	0.233	4.723	0.067	0.652
*N*	1479	1365	1365	1479	1479	998	998
Combined Effects:							
At will if no regular pay	0.069∗∗ (0.032)	−0.067∗∗ (0.034)	0.022 (0.039)	0.087∗∗ (0.035)	1.471∗ (0.840)	0.041∗ (0.024)	0.498 (0.313)
Monthly if no regular pay	−0.026 (0.034)	0.012 (0.036)	−0.012 (0.040)	0.002 (0.037)	−0.128 (0.878)		

Note: Controls in all regressions include: high subsidy and deposit interventions, pre-intervention outcomes (mechanized desludging in year before baseline and manual desludging in year before baseline), unbalanced controls (hhd wealth and hhd size), and fixed effects at the grid-point level. Standard errors are in parentheses: p∗<0.10, p∗∗<0.05, p∗∗∗<0.01. Having regular employment is measured in the baseline. Outcome variables are (1) purchased the subsidized mechanized desludging through the subscription, (2) purchased an unsubsidized mechanized desludging between the baseline and endline, (3) purchased any mechanized desludging between the baseline and endline, (4) used the desludging account, (5) total value deposited in the desludging account (in thousands of CFA, 1000 CFA is approximately $2), (6) number of non-final deposits, and (7) value of non-final deposits (in thousands of CFA). In columns (1) through (5) the excluded treatment group control variable is pay in full. Columns (6) and (7) drop the pay-in-full group, and the excluded treatment group control variable is monthly billing. In the lower panel, ‘At will if no regular pay’ shows the value of the sum of the coefficient on ‘Save at will’ and ‘Save at will x No regular pay,’ the heterogeneous treatment effect.

Table 6 explores heterogeneous impacts of the subsidy level and deposit requirement in the full sample. Column (1) shows that in terms of signing up, there is not a significant difference in the effect of the subsidy and deposit requirement on those with and without regularly paying jobs. Column (2) shows that the high subsidy has a larger effect on purchasing the subsidized desludging on those with a regular salary. The effect of the high subsidy on actual purchases of a mechanized desludging (in column (4)) are also stronger among those with a regular job. Overall, the effect of the subsidies on those with and without a job with a regular salary are similar, and if anything are larger for those with a regular paying job.

In Table 7 we look at the differential impact of the mobile money treatments on those with and without regular salaries. Individuals with a regular job have a regular source of money which they could potentially deposit into the save-at-will Wari account. But, since they do not experience as much volatility in their income flows, they may have less use for an account since they do not periodically have irregularly high income flows.

Conforming with our hypothesis that the ability to save whenever funds are available may be particularly important for those without a regular salary, we find that the effects of the mobile money treatment are particularly large for this group. Households in which the decider has no consistent monthly salary are 6.9 percentage points more likely to purchase the subsidized desludging when they have access to an account that allows them to deposit money whenever it is available. The save-at-will treatment causes these households to be 8.7 percentage points more likely to deposit money in the account, and deposit around 30% more in the account overall. We also see that the mechanism vis-a-vis which we suspect the save-at-will treatment impacts individuals continues to hold. Individuals without regular salaries are more likely to save over time; when they are in the save-at-will treatment they are 4.1 percentage points more likely to make non-final deposits in the account prior to their desludging than those in the monthly-billing group.

Next we look at heterogeneity with respect to the household's mechanized desludging history in Table 8, Table 9. The bottom panel of Table 8 shows that the high subsidy is very effective at convincing those who have purchased a mechanized desludging in the year before the baseline survey to switch from purchasing a mechanized desludging on the open market to purchasing one from our subsidized program. But, the subsidy does not affect these people's overall purchases of mechanized desludgings. These individuals were probably going to purchase a mechanized desludging no matter what, and the high subsidy merely convinces them to switch away from their usual provider to purchase through us instead.

**Table 8:** Heterogeneous impacts in the full sample: household had mechanized desludging in past year.

	(1)	(2)	(3)	(4)
	Signed Up	Subsidized Desludging	Unsubsidized Mechanized Desludging	Any Mechanized Desludging
High subsidy	0.213∗∗∗ (0.028)	0.057∗∗∗ (0.016)	−0.049∗∗ (0.024)	0.012 (0.025)
Deposit required	−0.093∗∗ (0.043)	−0.047∗∗ (0.024)	0.025 (0.036)	−0.032 (0.038)
Mechanized desludging in year before baseline	0.140∗∗ (0.062)	−0.030 (0.035)	0.483∗∗∗ (0.054)	0.459∗∗∗ (0.056)
Mechanized desludging more than a year before baseline	0.139∗ (0.081)	0.047 (0.045)	0.074 (0.070)	0.140∗ (0.073)
Never desludged before baseline	0.009 (0.064)	−0.040 (0.036)	−0.025 (0.055)	−0.076 (0.057)
High subsidy × Mechanized desludging in year before baseline	0.033 (0.040)	0.113∗∗∗ (0.022)	−0.115∗∗∗ (0.034)	0.000 (0.036)
High subsidy × Mechanized desludging more than a year before baseline	0.052 (0.052)	0.016 (0.029)	0.084∗ (0.044)	0.085∗ (0.046)
High subsidy × Never desludged before baseline	−0.102∗∗ (0.041)	−0.035 (0.023)	0.044 (0.036)	0.002 (0.037)
Deposit required × Mechanized desludging in year before baseline	−0.038 (0.061)	0.063∗ (0.034)	−0.027 (0.053)	0.036 (0.055)
Deposit required × Mechanized desludging more than a year before baseline	−0.085 (0.082)	−0.026 (0.045)	0.010 (0.070)	−0.024 (0.073)
Deposit required × Never desludged before baseline	0.022 (0.062)	0.068∗∗ (0.034)	−0.035 (0.053)	0.048 (0.055)
*R*^2^	0.252	0.194	0.356	0.416
Mean of Dependent Variable	0.398	0.074	0.237	0.315
*N*	3757	3757	3395	3395
Combined Effects:				
High subsidy if mech in year before baseline	0.246∗∗∗ (0.029)	0.170∗∗∗ (0.016)	−0.164∗∗∗ (0.025)	0.012 (0.026)
High subsidy if mech more than a year before baseline	0.266∗∗∗ (0.043)	0.072∗∗∗ (0.024)	0.035 (0.037)	0.097∗∗ (0.039)
High subsidy if never desludged before baseline	0.111∗∗∗ (0.030)	0.022 (0.017)	−0.004 (0.026)	0.014 (0.027)
Deposit if mech in year before baseline	−0.130∗∗∗ (0.043)	0.016 (0.024)	−0.003 (0.038)	0.004 (0.040)
Deposit if mech more than a year before baseline	−0.178∗∗ (0.069)	−0.073∗ (0.038)	0.034 (0.060)	−0.055 (0.062)
Deposit if never desludged before baseline	−0.070 (0.044)	0.021 (0.024)	−0.010 (0.039)	0.016 (0.040)

Note: Controls in all regressions include: pre-intervention outcomes (manual desludging in year before baseline) and fixed effects at the grid-point level. Standard errors are in parentheses: p∗<0.10, p∗∗<0.05, p∗∗∗<0.01. Outcome variables are (1) signed up for the subsidized mechanized desludging through the subscription, (2) purchased the subsidized mechanized desludging through the subscription, (3) purchased an unsubsidized mechanized desludging between the baseline and endline, and (4) purchased any mechanized desludging between the baseline and the endline. In the lower panel, ‘High subsidy if mech in year before baseline’ shows the value of the sum of the coefficient on ‘High subsidy’ and ‘High subsidy x Mechanized desludging in year before baseline,’ the heterogeneous treatment effect.

**Table 9:** Heterogeneous impacts in the subscriber sample: household had mechanized desludging in past year.

	(1)	(2)	(3)	(4)	(5)	(6)	(7)
	Subsidized Desludging	Unsubsidized Mechanized Desludging	Mechanized Desludging	Any Deposits	Value of Deposits	Any Non-Final Deposits	Value of Non-Final Deposits
Save at will	0.045 (0.059)	−0.088 (0.062)	−0.019 (0.070)	0.005 (0.065)	−0.251 (1.538)	0.010 (0.041)	0.224 (0.526)
Monthly billing	−0.034 (0.059)	−0.041 (0.061)	−0.062 (0.070)	−0.048 (0.064)	−0.707 (1.527)		
Mechanized desludging in year before baseline	0.051 (0.059)	0.300∗∗∗ (0.062)	0.372∗∗∗ (0.070)	0.036 (0.064)	1.961 (1.517)	−0.044 (0.043)	−0.473 (0.553)
Mechanized desludging more than a year before baseline	0.077 (0.071)	0.085 (0.073)	0.143∗ (0.083)	0.038 (0.078)	1.415 (1.840)	0.015 (0.047)	0.516 (0.599)
Never desludged before baseline	−0.018 (0.066)	−0.076 (0.067)	−0.072 (0.077)	−0.080 (0.072)	−1.327 (1.699)	0.006 (0.045)	0.275 (0.583)
Save at will × Mechanized desludging in year before baseline	0.042 (0.075)	−0.003 (0.079)	0.021 (0.089)	0.085 (0.082)	2.165 (1.951)	0.044 (0.053)	1.026 (0.682)
Save at will × Mechanzied desludging more than a year before baseline	−0.129 (0.099)	0.077 (0.103)	−0.030 (0.118)	−0.045 (0.108)	−0.614 (2.561)	0.041 (0.067)	−0.073 (0.863)
Save at will × Never desludged before baseline	0.023 (0.083)	0.039 (0.087)	0.032 (0.099)	0.085 (0.091)	1.548 (2.156)	−0.017 (0.057)	−0.387 (0.729)
Monthly billing × Mechanized desludging in year before baseline	0.104 (0.076)	−0.017 (0.079)	0.080 (0.090)	0.107 (0.083)	1.618 (1.968)		
Monthly billing × Mechanized desludging more than a year before baseline	−0.066 (0.094)	−0.021 (0.098)	−0.072 (0.111)	−0.007 (0.103)	0.050 (2.437)		
Monthly billing × Never desludged before baseline	−0.006 (0.083)	0.104 (0.085)	0.069 (0.097)	0.080 (0.090)	0.783 (2.143)		
*R*^2^	0.325	0.412	0.474	0.311	0.292	0.370	0.312
Mean of Dependent Variable	0.188	0.212	0.406	0.233	4.723	0.067	0.652
*N*	1479	1365	1365	1479	1479	998	998
Combined Effects:							
At will if mech in year before baseline	0.087∗ (0.045)	−0.091∗ (0.047)	0.002 (0.053)	0.089∗ (0.049)	1.914∗ (1.159)	0.054 (0.034)	1.250∗∗∗ (0.433)
At will if mech more than a year before baseline	−0.083 (0.078)	−0.011 (0.082)	−0.049 (0.093)	−0.041 (0.085)	−0.865 (2.016)	0.051 (0.054)	0.151 (0.691)
At will if never desludged before baseline	0.068 (0.057)	−0.048 (0.061)	0.013 (0.069)	0.089 (0.063)	1.297 (1.488)	−0.007 (0.041)	−0.162 (0.523)
Monthly if mech in year before baseline	0.070 (0.047)	−0.057 (0.049)	0.018 (0.056)	0.059 (0.051)	0.911 (1.218)		
Monthly if mech more than a year before baseline	−0.100 (0.072)	−0.062 (0.074)	−0.134 (0.084)	−0.055 (0.079)	−0.657 (1.861)		
Monthly if never desludged before baseline	−0.040 (0.059)	0.063 (0.060)	0.007 (0.069)	0.032 (0.064)	0.076 (1.519)		

Note: Controls in all regressions include: high subsidy and deposit interventions, pre-intervention outcomes (manual desludging in year before baseline), unbalanced controls (hhd wealth and hhd size), and fixed effects at the grid-point level. Standard errors are in parentheses: p∗<0.10, p∗∗<0.05, p∗∗∗<0.01. Outcome variables are (1) purchased the subsidized mechanized desludging through the subscription, (2) purchased an unsubsidized mechanized desludging between the baseline and endline, (3) purchased any mechanized desludging between the baseline and endline, (4) used the desludging account, (5) total value deposited in the desludging account (in thousands of CFA, 1000 CFA is approximately $2), (6) number of non-final deposits, and (7) value of non-final deposits (in thousands of CFA). In columns (1) through (5) the excluded treatment group control variable is pay in full. Columns (6) and (7) drop the pay-in-full group, and the excluded treatment group control variable is monthly billing. In the lower panel, ‘At will if mech in year before baseline’ shows the value of the sum of the coefficient on ‘Save at will’ and ‘Save at will x Mechanized desludging in year before baseline,’ the heterogeneous treatment effect.

The second row of the second panel shows that the treatment effects on those who have purchased a mechanized desludging in the past, but more than a year ago, are quite different. Those individuals are also convinced by the high subsidy to purchase a mechanized desludging through our system, but in their case this leads to an increase in purchases of mechanized desludgings more generally. These individuals were likely on the fence about whether to purchase a mechanized or manual desludging, but were convinced to choose the more sanitary method by the high subsidy. The subsidies have no impact on the desludging purchases made by individuals who have never had any desludging of their current pit. These may be the individuals who have very well-made high-functioning pits which fill up much more slowly.

Table 9 looks at the heterogeneous impacts of the mobile money treatment groups based on the same sources of heterogeneity. Here the bottom panel shows us that the save-at-will treatment has an impact on only one sub-group: those who have had a mechanized desludging in the year before the baseline. For these individuals, having the opportunity to save at will makes them more likely to purchase a mechanized desludging through us, but less likely to purchase one on the open market. This is also the group that is most likely to take advantage of the opportunity to make non-final deposits. Thus we see that for sub-groups that were planning on purchasing a mechanized desludging anyway, the save-at-will option is appealing and they sort out of the open market and into using it. But, the earmarked mental accounting savings option does not convince people who were not going to purchase a mechanized desludging in the first place to switch from less sanitary methods.

## 7 Conclusion

Health and sanitation services have large externalities and therefore governments often consider subsidizing their purchase. While subsidies may be effective, increasing take-up through less expensive means such as expanding payment options could also help to improve welfare at lower budgetary cost. Mental accounting theories suggest that nudges such as earmarked accounts with monthly reminders and pre-paid deposits may be effective in increasing households’ likelihood of saving for the service over time. As mobile payment systems become more readily available, governments and firms can more easily implement such nudges. We test methods of relaxing the household budget constraint (subsidies) and behavioral nudges (earmarked savings accounts and pre-paid deposits) on take-up of our subscription mechanized desludgings and mechanized desludgings in general.

We find that, as expected, subsidies increase take-up of mechanized desludgings. Households offered large subsidies are eight percentage points more likely to purchase a desludging through our program and three percentage points more likely to purchase a mechanized desludging overall. While this is a statistically significant increase in take-up, such subsidy programs are expensive: high subsidies were $14 per desludging more than low subsidies, and households who purchase the mechanized desludging receive the subsidy whether or not they already planned to purchase a mechanized desludging.

Pre-paid deposit requirements have no statistically significant impact on the use of mechanized desludgings. Deposits can be useful in helping a program plan necessary procurement and expenses. However, one might be concerned that deposits would decrease take-up through limiting purchases to those who sign up when the product is initially offered. We find that while deposits do decrease the number of households who sign up for the service–households required to pay a deposit were 10 percentage points less likely to sign up–the households that decline to pay a deposit are those who would not have purchased anyway. Overall purchases of program mechanized desludgings and any mechanized desludgings are not statistically significantly different between households asked to deposit and households who were not.

We find a relatively small (five percentage points) but significant increase in take-up of program desludgings among the households with the save-at-will treatment. Such an intervention could be scaled up relatively inexpensively by a utility company or other organization interested in facilitating mechanized desludging services, increasing the ability of households to pay for the desludging services when they need it. This could provide a competitive edge: offering a flexible mobile savings feature could help companies steal clients from other companies and increase purchases of their product. Companies are not doing this yet, but as mobile money fees continue to go down and record-keeping ability continues to go up, we may see more of this in the future.

While the flexibility of being able to save in advance through the save-at-will treatment increased take-up through the program, the more rigid-seeming monthly-billing system did not increase demand for program desludgings. This is somewhat surprising given that monthly billing is closer to programs commonly used by utilities in the US to help consumers smooth payments, but highlights the value of flexibility to consumers.

When encouraging more sanitary but more expensive technologies, a policy-maker can decide between traditional policies such as subsidies, and mental accounting nudges such as pre-paid deposits and earmarked savings accounts with monthly reminders. In our setting, the high subsidy does encourage individuals to switch from less sanitary to more sanitary techniques. But, the mental accounting nudges are less successful; the pre-paid deposits have no effect and monthly billing has little impact on deposits and take-up of desludging. Being given access to an earmarked savings account does encourage individuals to switch away from their usual mechanized desludging provider and purchase the mechanized desludging through our program using the account, so it does offer appealing benefits to consumers. But, it does not lead people to change the desludging method (mechanized versus manual) that they use.

## Figures and Tables

**Table C-1:** Randomization balance - subscribers only.

	Mean (SD)	Coefficient (SE)			
	(1) Pay in full (489 Obs.)	(2) Save at Will (SW - 497 Obs.)	(3) Monthly Billing (MB - 510 Obs.)	(4) p-values SW = MB = 0	(5) Total Obs.
High subsidy	0.63 (0.48)	−0.019 (0.04)	−0.0043 (0.04)	0.85	1496
Deposit required	0.86 (0.35)	−0.053∗∗ (0.03)	−0.061∗∗ (0.03)	0.050	1496
Courtyard looks clean	0.81 (0.39)	−0.0087 (0.03)	0.032 (0.03)	0.30	1479
Respondent years of education	6.75 (5.85)	0.16 (0.40)	0.22 (0.41)	0.86	1493
Respondent has no formal education	0.31 (0.46)	0.0098 (0.03)	0.014 (0.03)	0.91	1496
Respondent age	49.9 (13.13)	−0.21 (0.95)	−0.60 (0.96)	0.82	1482
Respondent male	0.70 (0.46)	−0.0067 (0.03)	−0.040 (0.03)	0.44	1496
Household size	9.93 (5.33)	0.22 (0.39)	0.99∗∗ (0.40)	0.034	1479
Number of rooms in house	6.88 (3.85)	−0.18 (0.26)	0.18 (0.26)	0.38	1479
Own their house	0.81 (0.40)	−0.026 (0.03)	0.012 (0.03)	0.39	1479
House has two stories	0.31 (0.46)	−0.036 (0.03)	0.017 (0.03)	0.24	1496
Wealth index	0.31 (1.51)	0.11 (0.11)	0.24∗∗ (0.11)	0.11	1479
Respondent has no regular pay	0.73 (0.44)	0.015 (0.03)	−0.016 (0.03)	0.62	1496
Used mechanized in year before baseline	0.40 (0.49)	−0.011 (0.03)	−0.052 (0.03)	0.25	1496
Used manual in year before baseline	0.29 (0.45)	0.021 (0.03)	0.078∗∗ (0.03)	0.053	1496
Used mechanized more than a year before bl	0.14 (0.35)	−0.025 (0.03)	0.022 (0.03)	0.20	1496
Never desludged before baseline	0.25 (0.43)	0.00076 (0.03)	0.013 (0.03)	0.88	1496
Responded to endline survey	0.92 (0.27)	−0.0045 (0.02)	−0.0064 (0.02)	0.94	1496
*p*-value of joint test		0.473	0.001	0.006	

Note: All variables are measured in the baseline (bl). Column (1) shows the mean and standard deviation in the pay-in-full group. Columns (2) and (3) show the coefficient on save at will and monthly billing in a regression including grid-point level fixed effects. The omitted treatment group in the regression is pay in full. Standard errors are in parentheses: p∗<0.10, p∗∗<0.05, p∗∗∗<0.01 in columns (2) and (3). Column (4) shows the *p*-values for tests of whether the coefficients in columns (2) and (3) equal one another and equal 0. The last row shows the *p*-values for a joint test of all the individual tests in the preceding rows.

**Table C-2:** Summary Statistics

Variable	Full		Subscriber	
	Sample		Subsample	
	Mean	Std Dev	Mean	Std Dev
**Outcome Variables**				
Signed up for subsidized mechanized desludging	0.398	0.490	1.000	0.000
Used subsidized mech desludging between bl and el	0.074	0.262	0.186	0.390
Got unsubsidized mech desludging between bl and el	0.237	0.425	0.212	0.409
Got any mech desludging between bl and el	0.315	0.465	0.405	0.491
Deposited in desludging account			0.231	0.422
Desludging account deposits (1000s of CFA)			4.683	9.854
Made non-final deposits (exclude pay-in-full group)			0.067	0.249
Non-final deposits (1000s of CFA) (exclude pay in full)			0.646	3.063
Days from interview to 1st desludging			128	102
Days from interview to 1st deposit			111	98
Days from 1st deposit to 1st desludging			14	49
Days from last deposit to 1st desludging			3	16
**Treatment Variables**				
High subsidy	0.503	0.500	0.626	0.484
Deposit required	0.875	0.330	0.840	0.367
Save at will			0.332	0.471
Monthly billing			0.341	0.474
Pay in full			0.327	0.469
**Other Baseline Variables**				
Courtyard looks clean in baseline	0.754	0.430	0.812	0.391
Respondent years of education	5.737	5.659	6.724	5.832
Respondent has no formal ed	0.384	0.486	0.313	0.464
Respondent age	49.58	13.56	49.48	13.17
Respondent male	0.672	0.470	0.710	0.454
Household size	10.25	5.81	10.29	5.63
Number of rooms in house	6.499	3.527	6.803	3.628
Own their house	0.778	0.416	0.815	0.389
House has two stories	0.242	0.429	0.301	0.459
Wealth index	0.008	1.588	0.346	1.611
Respondent has no regular pay	0.789	0.408	0.735	0.442
Used mechanized in year before baseline	0.294	0.455	0.364	0.481
Used manual in year before baseline	0.372	0.483	0.330	0.470
Used mechanized more than a year before baseline	0.128	0.334	0.144	0.352
Never desludged before baseline	0.271	0.445	0.243	0.429
*N*	3757		1496	

Summary statistics for the full sample and for subscribers only. Abbreviations bl and el represent baseline and endline respectively.

## Appendix A Script explaining subscription in surveys

*The below script is translated from Wolof to English. It appeared on the portable devices used by the enumerators with the different wordings automated by treatment group*.

Today, we are going to offer you a subscription to mechanized desludging. Mechanized desludging, it's very important, for you, your family, and your neighbors. When you use a truck to desludge your pit, the truck takes all of the filth from the pit, takes it far away from the house, so that you're sure that your house and the area around it is all very clean, and your children and other children in the neighborhood will not play in that filth.

The subscription that we're offering, it is very useful: it will help you plan for when you will need to desludge your pit, and it is thanks to the subscription that we will be able to subsidize the cost of a desludging over time, and it will enable you to have access to a quality desludging.

If you agree to subscribe, when you need a desludging, you will call ZZ, identify yourself as a subscriber, and say that you need a desludging. We'll then find a truck to desludge your pit within about 2–3 h of the call. The subscription for desludging includes 1 truck, which will do 1 trip, getting about 8 m^3^ from the pit, without ‘curage.'

Of the twelve houses that we chose to participate in the research, ten will be offered subsidized mechanized desludging. There are small subsidies and large subsidies, and of those 10 households, each has a 50% chance to have a large subsidy. The other two households will not be offered the chance to subscribe to the desludging service. We randomly selected the households' subsidies and wrote them on a piece of paper that I'll leave with you, but the amount of the subsidy for each household will not be known to the other households. *[Alt: We randomly selected the households' subsidies and wrote them on a piece of paper. I will list to you the subsidy of each household, and when we're done, I'll leave the list with you.]*

[*Enumerator: Pause, give the list to the respondent, and read it aloud with him.*]

You can use the subsidy twice to desludge your pit, within the next 9 months. [*Note: Later changed to 12 months.*] If you need more than two desludgings within that period, these additional desludgings will not be subsidized. Also, if you do not desludge your pit twice during this period, you will not be able to use the subsidy after those 9 [*Note: Later 12*] months.

In a few weeks, we will come back to the households that decide to sign up for the subscription to put on their door a sticker signaling that the house is a subscriber.

The undiscounted price of a desludging is 25000 fcfa. Your discount is: [discval]. So, you will pay [25000 - discval] for each of your first 2 desludgings over the next 9 months.

If you would like to sign up for a desludging, you will have to leave a deposit of 3000 fcfa. We will take this 3000 fcfa from your participation fee, so you will not have to give us any money out of pocket if you sign up. Would you like to sign up? [*Alt: Would you like to sign up for a mechanized desludging? You do not have to pay anything now.*]

[*Enumerator records whether the respondent signed up. The rest of the script is only read to subscribers.*]

The total remaining you will have to pay for a desludging is 25000 minus your discount of [discval]. [*Alt addition: You have made an initial deposit of 3000, that will be applied to the first desludging.*]

[*For save at will and monthly billing.*] If you wanted to save money for nine months to get your two desludgings by the end, you would have to save roughly XX each month.

[*For pay in full.*] If you wanted to get both desludgings in 9 months, you would pay XX for the first desludging and XX for the second desludging.

Wari is an option to save, receive, and send money through the telephone. Wari service points are all over Dakar: some are in banks or other places like PAMECAS; others are in places like Total gas stations, cybercafes, or corner boutiques. It is easy to identify these points because of the Wari sign on the door.

To deposit money in your Wari account, you can go to a Wari point, bringing your ID card and your Wari account number that I will give you. In order to deposit money you will pay a small fee: 100 fcfa for a deposit less than 5000 fcfa, and 2% if the deposit is 5000 fcfa or more.

When you deposit money, you will receive a message that confirms that you deposited. To withdraw money as well, meaning take part of your money out, you'll have to call this phone number: ZZ, and they'll tell you how it works. You can call this number from Monday to Friday between 8 a.m. and 6 p.m.

We will sign you up for a Wari mobile money account that you will use for your desludging.

[*For save at will.*] Whenever you want, you can deposit small or large amounts of money in your Wari desludging account. When you are ready for your desludging and you have enough money in your Wari account you can call our phone number to order the desludging. We will send you a text message every month to remind you how the account works.

[*For monthly billing.*] Every month we will message you a bill telling you how much you owe for your monthly payment of expected desludging expenses. When you are ready for your desludging and you have enough money in your Wari account you can call our phone number to order the desludging. We will send you a text message every month to remind you how the account works.

[*For pay in full.*] When you are ready for your desludging, you can deposit the full fee in your Wari desludging account and then call our phone number to order the desludging. We will send you a text message every month to remind you how the account works.

[*The script then asks the respondent details necessary to set up the Wari account such as his official name, phone number, id number, and address.*]

You must leave a deposit of 3000 fcfa, which will come from the participation fee that we discussed earlier. Your desludging Wari account now has 3000 fcfa in it. Any additional deposits can be made directly at Wari points in Dakar. Your 3 closest Wari points are: [Waript1, Waript2, Waript3] [*Enumerator, please write 3000 for the initial value of the desludging Wari account and give the card to the respondent.*] [*Alt: You don't have to deposit any money towards the desludging now, but you can go deposit money directly at Wari points anywhere in Dakar. Your 3 closest Wari points are: [Waript1, Waript2, Waript3]. [Enumerator, please write 0 for the initial value of the desludging Wari account and give the card to the respondent.]*]

Please take good care of this card. When depositing or withdrawing, you will need to give this account number. If you lose the card, you may call the number on your consent form to request that we send you the number. Be aware that no one will be able to deposit to or withdraw from the account without presenting the ID card of the person/people whose name is associated with the account. Also know that all transactions on this account will be confirmed with texts to the phone number associated with the account.

## Footnotes

1 In a developed country context, this is similar to rural households needing to have their septic tank emptied periodically. In developing countries this is an issue in urban areas as well as rural areas.

2 Of those individuals in our survey who have had their current pit fill up at least twice, 79% of them say this happens at least once a year.

3 Wari wanted to work with us partially as an experiment to see if savings accounts were a business they wanted to get involved in more generally. After our intervention ended, they decided to return any money left in our subscribers' savings accounts and not continue offering such products.

4 These are the fees Orange Money told our Senegalese project staff they were charging in mid-2017. We have been unable to obtain official information on Orange Money's fee structure in 2014, but in personal communications our project staff suggested that fees in the past were higher than the more recent 6%.

5 Coming out the door of the house closest to the grid-point, the teams would turn right, mapping households on both sides of the street, and turning right at every corner. If one circuit of the block was not enough to identify 25 households, they would return to the original household and spiral out to take the second right instead of the first right.

6 The additional two households are used to measure spillovers described in Deutschmann et al. (2018).

7 In Deutschmann et al. (2018), we analyze the effects of the different saturation levels across neighborhoods.

8 Procurement was through first-price just-in-time auctions conducted with desludging operators using SMS messages, a process that is the topic of a separate research paper, Houde et al. (2018).

9 Following the intervention period, the procurement system was handed over to the government of Senegal's sanitation department which continues to run the system for procurement purposes (but not the subscription program).

10 Of those who did not sign up for the service, 31% stated that the primary reason they did not sign up was that the price was still too high, 28% stated that the primary reason was that they thought they would not need a desludging in the near future, and 23% stated that they could not sign up without first consulting other family members.

11 Because of the distributed nature of Wari's service network, and the fact that savings accounts were new for Wari, some clients reported service points which did not know how to deposit money in their accounts. We worked with Wari and directly with operators to resolve issues when they were reported, but many may have gone unreported at the time.

12 Simple *t*-tests of the means of save at will versus pay in full, pay in full versus monthly billing, and save at will versus monthly billing give *p*-values of 0.29, 0.23, and 0.94 respectively. If one runs a regression with lack of understanding on the left-hand side and with controls as in Table 3 on the righ-hand side, the coefficients (standard errors) on save at will and monthly billing are 0.02 (0.08) and 0.03 (0.07) respectively. When testing that the two equal one another, the *p*-value is 0.93.

13 Inflation in Senegal over this time period was slightly negative, so this should not act as a deterrent to saving.

14 Individuals in the pay-in-full group were not supposed to be able to make non-final deposits, but one person did so before Wari successfully enforced that restriction.
